# Replication stress affects the fidelity of nucleosome-mediated epigenetic inheritance

**DOI:** 10.1371/journal.pgen.1006900

**Published:** 2017-07-27

**Authors:** Wenzhu Li, Jia Yi, Pamela Agbu, Zheng Zhou, Richard L. Kelley, Scott Kallgren, Songtao Jia, Xiangwei He

**Affiliations:** 1 Life Sciences Institute and Innovation Center for Cell Signaling Network, Zhejiang University, Hangzhou, Zhejiang, China; 2 Department of Biochemistry and Molecular Biology; 3 Department of Molecular and Human Genetics, Baylor College of Medicine, Houston, TX, United States of America; 4 Department of Biological Sciences, Columbia University, New York, NY, United States of America; The Babraham Institute, UNITED KINGDOM

## Abstract

The fidelity of epigenetic inheritance or, the precision by which epigenetic information is passed along, is an essential parameter for measuring the effectiveness of the process. How the precision of the process is achieved or modulated, however, remains largely elusive. We have performed quantitative measurement of epigenetic fidelity, using position effect variegation (PEV) in *Schizosaccharomyces pombe* as readout, to explore whether replication perturbation affects nucleosome-mediated epigenetic inheritance. We show that replication stresses, due to either hydroxyurea treatment or various forms of genetic lesions of the replication machinery, reduce the inheritance accuracy of CENP-A/Cnp1 nucleosome positioning within centromere. Mechanistically, we demonstrate that excessive formation of single-stranded DNA, a common molecular abnormality under these conditions, might have correlation with the reduction in fidelity of centromeric chromatin duplication. Furthermore, we show that replication stress broadly changes chromatin structure at various loci in the genome, such as telomere heterochromatin expanding and mating type locus heterochromatin spreading out of the boundaries. Interestingly, the levels of inheritable expanding at sub-telomeric heterochromatin regions are highly variable among independent cell populations. Finally, we show that HU treatment of the multi-cellular organisms *C*. *elegans* and *D*. *melanogaster* affects epigenetically programmed development and PEV, illustrating the evolutionary conservation of the phenomenon. Replication stress, in addition to its demonstrated role in genetic instability, promotes variable epigenetic instability throughout the epigenome.

## Author summary

In this study, we found replication stresses reduce the fidelity of nucleosome-mediated epigenetic inheritance. Using Position Effect Variegation (PEV) in centromere as an indicator of chromatin epigenetic stability, we quantified the precision of nucleosomal inheritance and found replication stresses reduce the fidelity of nucleosome-mediated epigenetic inheritance. Further analysis of genome-wide heterochromatin distribution showed that replication stresses affect chromatin structure by expanding of heterochromatin with locus specificity. Mechanistically, we provide evidence suggesting that excessive formation of single-stranded DNA might have correlation with the reduction in fidelity of centromeric chromatin duplication. Finally, we demonstrated replication stress perturb the development process by reducing the fidelity of chromatin organization duplication in fruit fly and worm, illustrating the broadness and the evolutionary conservation of the phenomenon. Together, our results shed light on the importance of replication stresses cause epigenetic instability in addition to genetic stability.

## Introduction

In eukaryotic cells, genomic DNA are packaged into arrays of nucleosomes [1], each comprised of a 147bp DNA fragment wrapped around a histone octamer core. The combination of histone variants and the large repertoire of covalent modifications on histones result in a highly complex biochemical signature of the nucleosome, which encodes important epigenetic information [2,3]. Overall, the nucleosomal organization of chromatin–including the positions of nucleosomes relative to the underlying DNA sequence and the biochemical signatures that they carry–has a profound impact on the functional state of the genome. In order to maintain the identity of the cell, nucleosomal organization must be preserved through cell divisions. On the other hand, it is conceivable that controlled alteration in cell type, such as cell differentiation during development, would require nucleosomal organization amendable for reprogramming. Despite its profound biological significance, the mechanisms on regulating or influencing the precision of chromosomal epigenetic inheritance are not well understood. In this study, we examine the effect of replication perturbation on the fidelity of chromatin duplication and epigenetic inheritance and explore the underlying mechanisms.

During cell division, chromatin is duplicated in conjunction with DNA synthesis at the replication fork, through a process called replication coupled (RC) nucleosome assembly [4,5]. The process can be divided into three major steps. First, pre-existing nucleosomes (also known as parental nucleosomes) immediately in front of the replication fork are disrupted so that the template DNA is accessible to the replication machinery. Second, shortly after replication fork passage, the core components of the parental nucleosomes–the (H3-H4)_2_ tetramers in specific–are recycled to assemble nucleosomes on one of the daughter strands behind the replication fork. Finally, newly synthesized histones are incorporated on the other daughter strand to form nucleosomes *de novo*. Although the two daughter strands can be distinguished based on whether they originate from the leading or lagging strand in replication, in general, there appears to be no strand-specificity for histone recycling and *de novo* nucleosome assembly.

To achieve precision in duplication of epigenetic markers on histones, the recycled parental H3-H4 molecules need to be incorporated at their original loci on one of the daughter strands. Furthermore, the nucleosomes assembled *de novo* on the other strand need to be positioned at the corresponding sites. The newly incorporated histone molecules should also be of the proper variant type and obtain the biochemical modifications matching that of the parental histones. Currently, little is known on how the precision is achieved for any of the above steps. An important aspect of chromatin duplication is that the parental H3-H4 histones are transferred from the template chromatin to the replicated strands as intact (H3-H4)_2_ tetramers [6–8]. An alternative mode of inheritance–splitting the (H3-H4)_2_ tetramer into two dimers and passing them equally to the two replicated strands–may occur in the minor sub-population of nucleosomes that contain the H3.3 variant but not in the majority that contain the canonical H3.1 variant [8].

The DNA replication machinery is directly implicated in RC-nucleosome assembly by interacting with, and potentially coordinating the actions of, histones, histone chaperones, histone modifying enzymes, and chromatin remodeling factors[9,10]. Among the replication proteins, the helicase MCM2-7 is thought to play critical roles in evicting the nucleosomes ahead of the replication fork as well as assembling nucleosomes behind the fork. MCM2-7 forms a stable complex with the histone chaperone Asf1 that is bridged by a histone H3-H4 dimer [11,12]. The Asf1/H3-H4/MCM complex may represent an intermediate for parental histone recycling or new nucleosome assembly. However, it remains unclear how each (H3-H4)_2_ tetramer is integrated into the proper site on one of the daughter strands. In the budding yeast *S*. *cerevisiae*, genome-wide tracking of the parental histone H3 molecules through several generations and quantitative modeling of experimental data lead to a model that the parental (H3-H4)_2_ tetramers are re-incorporated within the distance of one to two nucleosomes (~400bp) of the original site. Thus, nucleosomal inheritance may be somewhat “sloppy” [13]. Such sloppiness, if confirmed, would preclude single or small number of nucleosomes as efficient carrier of epigenetic information. Further experimental evidence is needed to directly test this model.

We used Position Effect Variegation (PEV) as an indicator of chromatin epigenetic stability to quantify the precision of nucleosomal inheritance. PEV, referring to variable expression patterns in a gene due to its translocation to a specific position in the genome, was broadly observed [14,15]. PEV phenomenon was originally discovered in specific fruit fly strains, associated with the *white* gene translocation adjacent to centromeric heterochromatin. There, the variegated expression states of the translocated *white* gene propagate clonally in adult eyes, causing mosaic eye coloration patterns [14]. In fission yeast, heterochromatin spreading is also responsible for PEV associated with the mating type locus. Within the centromeric core region, PEV is also observed but due to a different mechanism [16,17]. Here, the positioning of nucleosomes containing the specific histone H3 variant CENP-A/Cnp1 is variable within the centromere, and Cnp1 occupancy inversely correlates with the expression levels of the underlying reporter genes.

Regardless of the biochemical characteristics of local chromatin region responsible for underlying gene silencing, one common property of PEV is that the variegated gene expression states are inherited in a clonal fashion. In both budding yeast and fission yeast, inheritance of variegated gene expression is vividly demonstrated by sectoring in yeast colony coloration [16,18,19] and by tracking the gene expression status through the cell generations directly at single cell level [16]. Changes in the coloration patterns of the colonies serves as a convenient indicator of changes in the epigenetic marker underlying gene silencing.

In addition to using PEV as readout for epigenetic stability at specific loci, we wish to assess the impact of replication stresses at the whole epi-genome level by determining the genome-wide heterochromatin distribution in cells under stresses. Finally, we sought to explore the possible evolutionary conservation and the physiological significance of reduced epigenetic fidelity due to replication stress by testing the effects of perturbing replication on the development process in fly and worm.

## Results

### Hydroxyurea treatment enhances centromeric PEV in *S*. *pombe*

In fission yeast, variegated expression of *ade6*^*+*^ inserted in the centromere (*cnt2*::*ade6*^*+*^) is readily visualized: the ON or OFF states of *ade6*^*+*^ correspond to white or red color, respectively, of the colonies grown with low supply of adenine. We previously have shown that cnt2::*ade6*^*+*^ expression inversely correlates with Cnp1 occupancy on *ade6*^*+*^. Furthermore, by tracking the colony coloration through cell lineages, we have demonstrated that the state of *ade6*^*+*^expression, and thereby, Cnp1 occupancy on *ade6*^*+*^, is inherited through cell generations, but can change abruptly within one generation at low rates [16].

**Fig 1:**
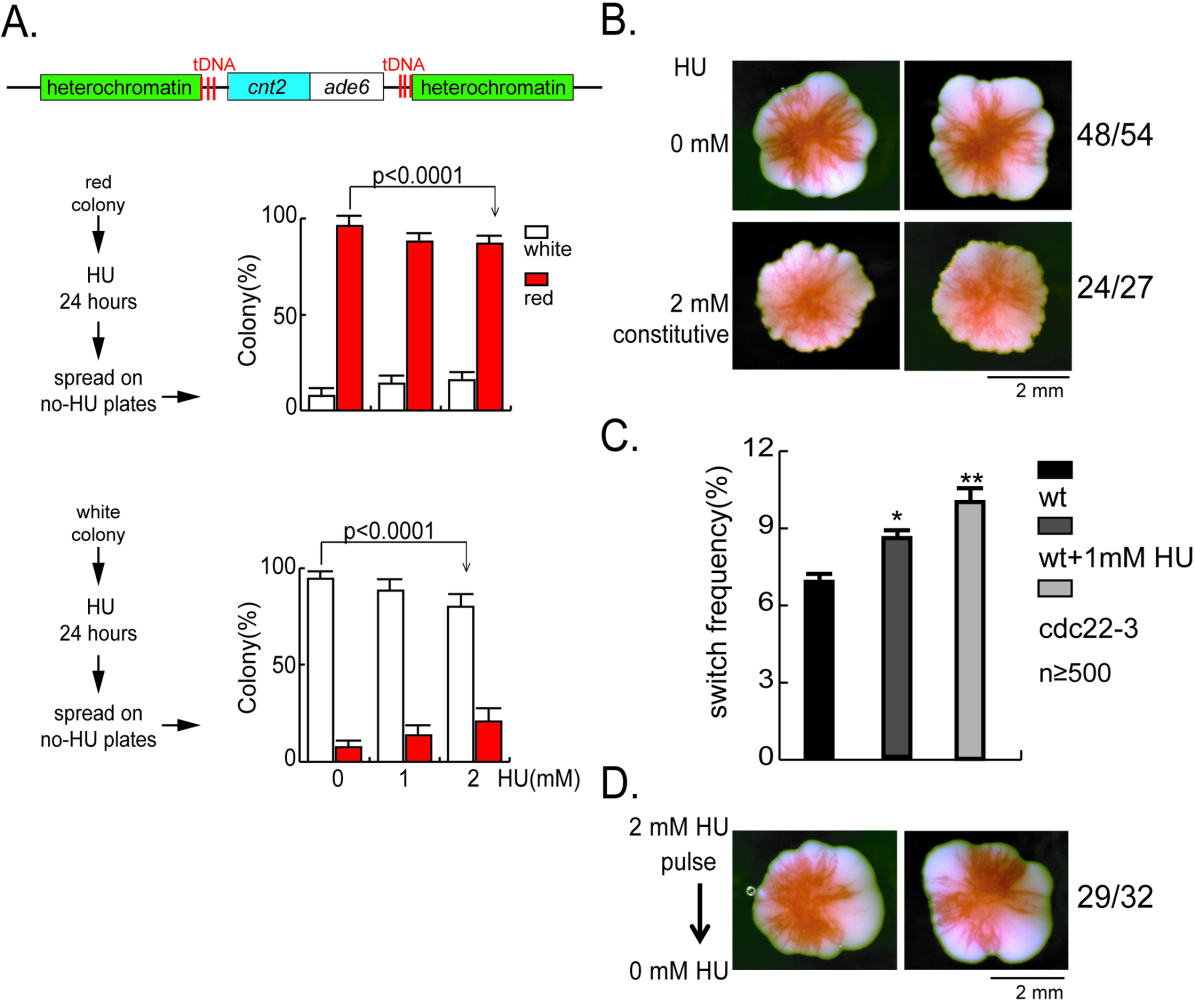
Hydroxyurea (HU) treatment enhances centromeric PEV. **(A)** HU promotes switching in expression states of *cnt2*::*ade6*^+^ in a dosage-dependent manner. Cell suspensions of a red (upper panel) or a white (lower panel) colony are grown in liquid media with HU as labeled for 24 hours, washed and spread onto YE+4S (low adenine) plates, with >500 cells/plate. The red or white colonies are counted at 5th day when incubated at 25°C. Each experiment has been performed by at least triple biological repeats. Each biological repeat has its unique composition of red/white cell ratio in the initial culture. One biological experiment result is shown here. The error bars are 1 SD of percentages for four plates. *p* values are calculated by Fisher exact test. **(B)** The red colony exhibits enhanced sectoring pattern upon HU treatment. Cells derived from the above red colony (before HU treatment) are planted evenly at one cell/cm^2^ by microscopic manipulation on YE+4S plates containing HU as labeled, incubated at 25°C for eight days. Two representative colonies for each are shown. The number shown here means the number of red colonies in the total colonies. **(C)** Elevated frequencies of *cnt2*::*ade6*^*+*^ red-to-white switching measured by pedigree analysis in wild type cells with HU treatment and *cdc22-3* mutants. Pedigree analyses are performed on “hybrid” plates for measuring switching rate of HU treated cells, each with a drug-free zone and a drug-containing zone. For each pedigree, three cell generations are tracked at the zone containing HU as labeled. The eight progeny cells are then moved to the drug-free zone, incubated at 25^°^C for five days. For each experimental condition (wild type with or without HU treatment and *cdc22-3* mutant), colony color switching events are scored in 400–1000 cell division events in total. The error bars are 1 SD of percentages for four independent experiments. **(D)** Colonies maintain the wild type level of sectoring after short-term HU treatment. Cell suspensions of a red colony in (A) grown under 2mM HU is washed and planted evenly at 1 cell/cm^2^ in YE4S media, and incubated at 25°C for eight days.

We explored the possible association between the fidelity of centromeric Cnp1 nucleosome position inheritance and the progression of DNA replication. Hydroxyurea (HU) is broadly used to study DNA damage-independent replication fork arrest [20–22]. HU is an inhibitor of ribonucleotide reductase (RNR), the enzyme responsible for the synthesis of dNTPs. Depletion of dNTP pools through HU treatment leads to replication fork arrest and subsequent genomic instability[23,24]. We first tested whether HU treatment enhances the rate of switching in the expression state of *cnt2*::*ade6*^+^. Yeast cells originated from a red colony (*i*.*e*., *cnt2*::*ade6*^*+*^ silenced, with minor white sectors) were treated transiently (for four to six generation times) with low concentrations of HU, prior to plating on the media for characterization of the progeny colonies coloration. Consistent with the epigenetic inheritance of the *cnt2*::*ade6*^*+*^ silent status, in the absence of HU, most of the progeny colonies were red (with minor white sectors). However, with increasing concentrations of HU, more white (with or without minor red sectors) colonies were formed (Fig 1A, upper). Similarly, HU treatment of cells that originated from a white colony also gave rise to increased switching in colony coloration, albeit from white to red (Fig 1A, lower).

We previously have established a pedigree analysis assay to quantify the rate of switching in *cnt2*::*ade6*^*+*^ expression states per cell division [16]. Using this assay, we measured the switching rate of *cnt2*::*ade6*^*+*^ (OFF to ON) in wild type cells is 6.5%, and is increased to 8.5% in wild type cells treated with 1mM HU (Fig 1B lower panel). These results show that HU treatment enhances switching in *cnt2*::*ade6*^*+*^ expression status, suggesting that replication perturbation due to HU treatment (which inhibits ribonucleotide reductase and causes depletion of dNTP pools) could reduce the fidelity of centromeric epigenetic inheritance. To test whether depletion of dNTP pools by genetic perturbation would have the same effect, we also measured the CEN-PEV switching rate in *cdc22-3*, a mutation in the large subunit of ribonucleotide reductase[25]. The result shows that the switching rate of *cnt2*::*ade6*^*+*^ (OFF to ON) in *cdc22-3* at 25^°^C is increased to 9.7% comparing with 6.5% in wild type cells (Fig 1B lower panel), suggesting that replication perturbation caused by suboptimal dNTP levels reduces the fidelity of centromeric epigenetic inheritance.

Likewise, colonies formed at the constant presence of HU exhibit higher degrees of sectoring than those formed in the absence of HU. This reflects enhanced, continually ongoing switching in *cnt2*::*ade6*^*+*^ expression state throughout the time course of colony formation (Fig 1B). Noticeably, colonies that exhibited high degree sectoring when grown in the presence of HU, once re-plated on media without HU, reverted to wild type degree sectoring (Fig 1C). This suggests that change in the degree of sectoring directly correlates with HU treatment and that such changes are not genetic. To further confirm this notion, we examined the *ade6* gene in four of these red colonies by PCR amplification and DNA sequencing and found no mutation. Together, these results indicate that HU-induced perturbation of DNA replication promotes switching in *cnt2*::*ade6*^*+*^ expression status, and once switched, the expression states are inherited.

### HU-induced enhancement of centromeric PEV correlates with excessive production of single strand DNA (ssDNA) in S phase

HU treatment disturbs replication progression as well as RC nucleosome assembly. Specifically, continuing unwinding template DNA combined with pausing in DNA synthesis leads to excessive formation of ssDNA on template and concurrent accumulation of parental H3-H4 histones evicted from template chromatin [26]. ssDNA activates the S phase checkpoint, which in turn, halts the MCM helicase, preventing further ssDNA formation and histone eviction [27,28]. When replication resumes, incorporation of the accumulated histones onto the daughter strands would be disordered due to the loss of their original positioning on the template chromatin [26], thus contributing to the enhanced variegation in the centromere.

**Fig 2:**
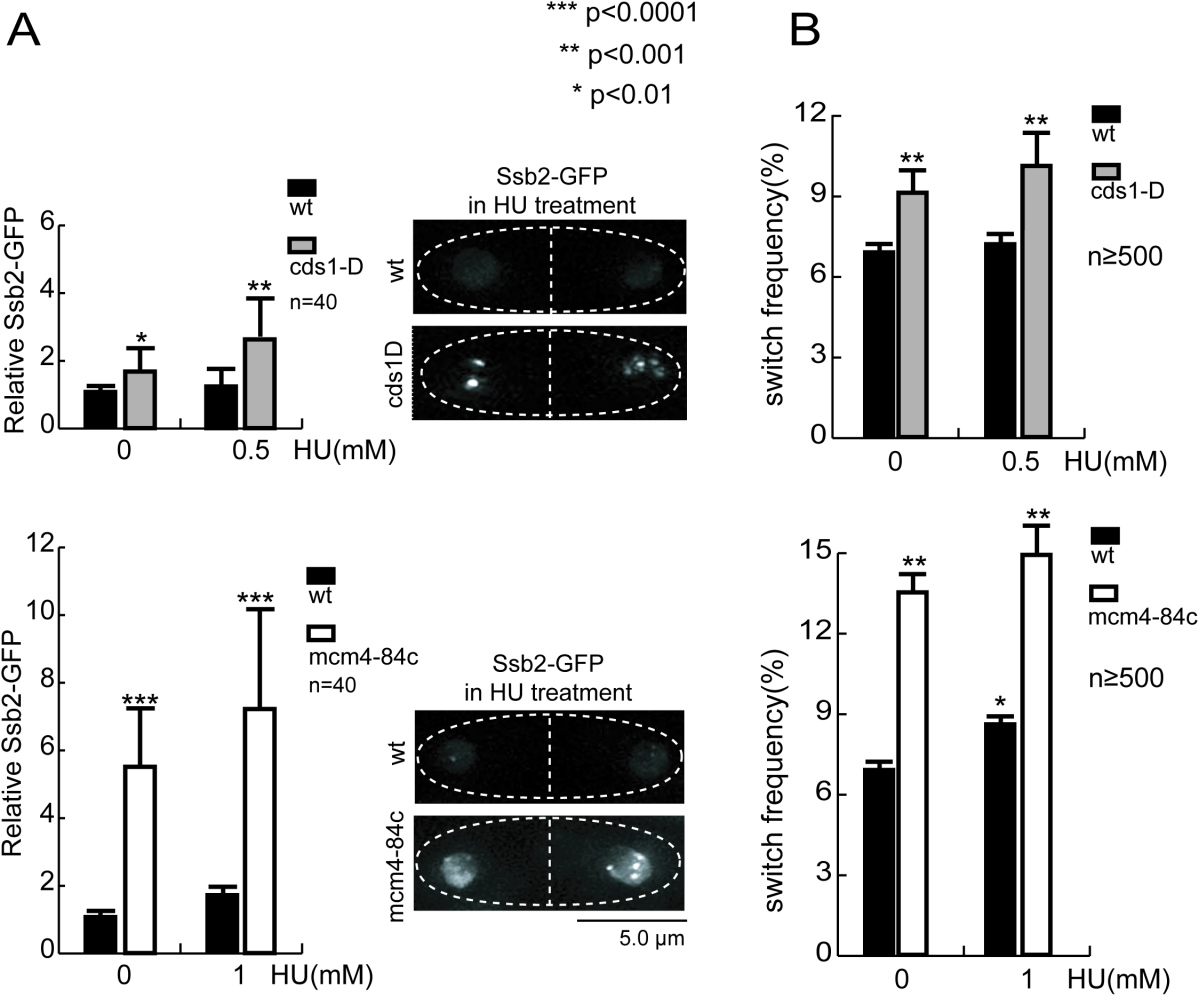
Enhanced centromeric PEV correlates with excessive formation of ssDNA upon HU treatment. **(A)** Quantification of Ssb2-GFP foci signal in S-phase cells. Cells are treated with HU as labeled for 6 hours, the Ssb2-GFP signal is quantified microscopically in live S phase cells (with a septum). Representative cell treated with HU images are shown on right. Ssb2-GFP signal in each nucleus equals the sum of all the foci within the nucleus. Forty nuclei are measured each and the average ± SD are shown, normalized with the value of wild type cells with no HU (1X). *p* values are calculated by t test. **(B)** Elevated frequencies of *cnt2*::*ade6*^*+*^ red-to-white switching measured by pedigree analysis in *cds1-D* and *mcm4-84c* mutants. Switching rate is measured as in Fig 1C. For each experimental condition (specific genotype plus HU concentration), colony color switching events are scored in 500–600 cell division events in total. The error bars are 1 SD of percentages for four independent experiments.

According to this model, there would be a correlation between excessive ssDNA formation and the reduction in the fidelity of nucleosome inheritance. In particular, mutations that cause prolonged unwinding of the template DNA and excessive formation of ssDNA should also reduce the fidelity of nucleosome inheritance. We chose two mutants to test this prediction: deletion of the S phase checkpoint gene *cds1 (rad53/chk2)*—*cds1-D* and a c-terminal truncation of the Mcm4 subunit of the MCM helicase -*mcm4-84c*. *cds1-D* inactivates the replication checkpoint [29,30]; whereas *mcm4-84c* renders the MCM helicase unresponsive to inhibition by an activated checkpoint without apparent compromising of its helicase activity [28]. Both mutants are hypersensitive to HU (S1 Fig), and are shown to form ssDNA excessively upon replication pausing as evidenced by enhanced chromatin association of single strand binding protein, Ssb2/Rfa2 [28,31]. We confirmed this by quantifying the Ssb2-GFP foci signal in S phase cells (recognized by the presence of a medial septum–a morphological signature of S phase cells). The Ssb2-GFP foci signal is significantly increased in both *cds1-D* and *mcm4-84c* mutants compared with wild type at the same HU doses (Fig 2A). In order to test whether the increased Ssb2-GFP signal results from the change in the Ssb2-GFP protein level, we have performed the western blotting assay, and found the similar protein level between wild type and mutant cells with or without HU treatment (S2 Fig).

We then compared the rate of switching in *cnt2*::*ade6*^*+*^ expression states upon HU treatment in wild type and mutant cells. In wild type cells, HU treatment enhances switching rate in *cnt2*::*ade6*^*+*^ expression status using the pedigree analysis assay [16]. Meanwhile, we measured significant increases in the rate of switching in *cds1-D* cells (9.1% without HU treatment to 10.1% treated with 0.5mM HU) and *mcm4-84c* cells (13.5% without HU treatment to 14.9% treated with 1mM HU) respectively (Fig 2B). Both mutants also exhibited higher rates of switching than wild type cells under all tested conditions. Consistently, we also found that mutant colonies exhibited more complex sectoring patterns with HU treatment compared to no HU treatment, and much more complex sectoring patterns in comparison to wild type at all conditions (S1 Fig). Together, these results suggest that excessive ssDNA formation caused by HU treatment is correlated with the reduction in the fidelity of epigenetic inheritance.

**Fig 3:**
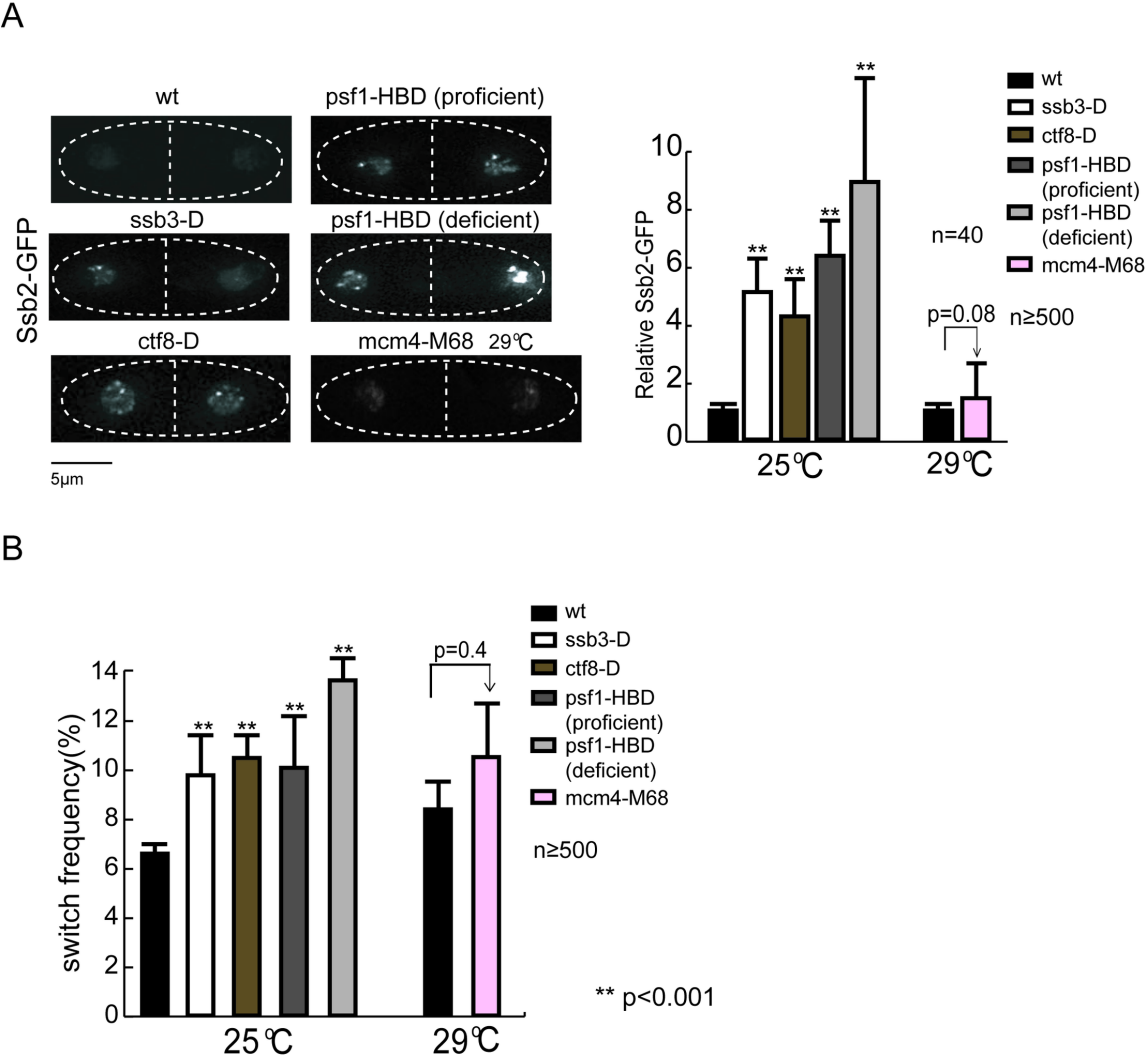
Mutations in the replication machinery enhance centromeric PEV correlates with excessive formation of ssDNA. **(A)** Increased levels of Ssb2-GFP signal in the mutant S phase cells. Cells are grown in the liquid rich media except for Psf1-HBD cells in YE+5S media plus 100nM β-estradiol (proficient condition) or 0.1nM β-estradiol (deficient condition), respectively. *mcm4-M68* cells are grown in 29^°^C for 6 hours. Ssb2-GFP signal is measured as in Fig 2A. Left panels, representative images of S phase cells. **(B)** Quantification of *cnt2*::*ade6*^+^ red-to-white switching frequency in the replication mutants. For *psf1*-HBD (deficient) cells, pedigree analyses are performed on “hybrid” plates, on which three cell generations are tracked at the “deficient”-zone containing 0.1nM β-estradiol. The eight progeny cells are then moved to the “proficient”-zone, containing 100nM β-estradiol, incubated at 25^°^C for five days. For *mcm4-M68* cells, pedigree analysis is performed at 29^°^C, and incubated at 25^°^C for 5 days before scoring. For each test, 500–800 cell division events are scored. The error bars are 1 SD of percentages for four independent experiments. *p* values are calculated by t test.

Conversely, if a mutation causes DNA replication stalling without excessive formation of ssDNA, it should not affect the fidelity of nucleosome inheritance. MCM helicase unwinds the template DNA and is postulated to drive the eviction of the parental histones. We thus reasoned that perturbation of the MCM helicase function might cause replication perturbation without causing excessive unwinding of template DNA or accumulation of parental histones. To test this, we examined the effect of a temperature sensitive mutation in the MCM helicase (*mcm4-M68*^*ts*^) that conditionally disrupts replication initiation [32]. At a semi-permissive temperature (29^°^C), the biological activity of MCM is compromised so that the survival of *mcm4-M68* cells is strictly dependent on the Chk1-dependent DNA damage checkpoint that is non-essential in wild type cells. Interestingly, the Cds1p-dependent intra-S phase checkpoint is not activated or required for cell survival under this stress condition [33]. Indeed, we found only insignificant level of Ssb2-GFP foci signal in S phase *mcm4-M68* cells at 29^°^C comparable to wild type (Fig 3A), but observed a significant cell cycle delay (elongated cell morphology) and reduction in cell viability (S3 Fig), confirming that MCM activity was compromised. Consistent with minor increase in ssDNA formation, the rate of switching in *cnt2*::*ade6*^*+*^ expression state increased insignificant in *mcm4-M68* cells compared to wild type at 29^°^C (Fig 3B). Mutant colony morphology also exhibited wild type level complexity in sectoring (S1 Fig).

Together, these results suggest a correlation between excessive ssDNA formation and the enhanced switching of centromeric PEV, indicating a reduction of fidelity in Cnp1 nucleosome position inheritance.

### Genetic perturbations of the replication machinery enhance centromeric PEV in correlation with excessive ssDNA formation

Given that HU treatment enhancing centromeric PEV correlates with excessive ssDNA formation, we sought to test whether other replication stresses that cause increased ssDNA formation should also cause enhanced centromeric PEV. We tested genetic perturbations in three distinct complexes of the replication machinery: deletion of *ctf8*, a non-essential subunit of the Ctf18-RFC clamp-loader complex [34]; and deletion of *ssb3*, a non-essential subunit of the ssDNA binding protein complex RPA [31], partial inactivation of Psf1, a subunit of the GINS complex essential for replication initiation and elongation [35]. Conditional inactivation of Psf1, an essential protein, is achieved by fusing Psf1 to a steroid hormone-binding domain (HBD) tag that is tightly associated with the protein chaperone Hsp90, rendering the fusion protein inactive by steric hindrance. The HBD fusion protein can be kept active by the addition of β-estradiol, which binds to HBD and displaces Hsp90. *psf1-HBD* cells depend on the presence of β-estradiol for viability [36].

Microscopic examination of S phase cells reveal increased Ssb2-GFP foci signal in all three mutants compared to wild type (Fig 3A). Noticeably, *psf1-HBD* cells with a high level of β-estradiol, which fully supported cell viability (“proficient” condition), still exhibit an elevated switching rate (10.4% in comparison to 6.5% in wild type) and enhanced ssDNA levels, indicating that the HBD tag alone may quantitatively disturb the GINS complex function. Reducing the level of β-estradiol (“deficient” condition) further exacerbate the defects (the switching rate increases to 13.9%). Consistently, quantification of the switching rate (*cnt2*::*ade6*^*+*^ OFF to ON) by pedigree analysis show higher switching rate in all mutants compared to wild type cells (Fig 3B), mutant colony morphology also exhibits more complex sectoring patterns (S1 Fig).

We further wish to test whether reduced epigenetic inheritance stability caused by replication stress is not only reflected by the enhanced switching rate of *cnt2*::*ade6*^*+*^ OFF to ON, but also the changed switching rate of *cnt2*::*ade6*^*+*^ ON to OFF. Consistently, the switching rate of *cnt2*::*ade6*^*+*^ in wild type cells is 6.5% (OFF to ON) and 3.4% (ON to OFF), and increased to 8.5% (OFF to ON) and 5.6% (ON to OFF) with 1mM HU treatment. And we also quantified the two switching rates of *cnt2*::*ade6*^*+*^ simultaneously in two replication mutants, *ssb3D and ctf8D*. While significant increases in switching rate of *cnt2*::*ade6*^*+*^OFF to ON were detected in both mutants (9.8% in *ssb3D* and 10.6% in *ctf8D*), no increase or slight reduction in ON to OFF rate was found (2.55% in *ssb3D* and 3% in *ctf8D*, respectively). We are unclear about the discrepancy between the measurements of the two switching rates. One possible reason may be that, the pedigree analysis is less suited to capture rare switching events (ON to OFF) quantitatively.

All together, in all three mutants tested above in which various parts of the replication machinery is perturbed genetically and with excessive accumulation of ssDNA, centromeric PEV is enhanced.

### Replication stress stimulates enhanced silencing of heterochromatin-dependent PEV

We further reasoned that replication stresses might affect the inheritance of other chromatin features in addition to Cnp1/CENP-A nucleosome occupancy within the centromeres. To test this idea, we examined two PEV systems associated with the mating type region caused by stochastic heterochromatin (histone H3K9me2/3 modifications) spreading.

The mating type region of fission yeast contains three gene loci, among which only *mat1* is actively transcribed and determines the cell mating type. *mat2* and *mat3* act as genetic information donors for a gene conversion process at *mat1* that causes mating type switching [37]. *mat2-mat3* region, in wild type cells, is silenced tightly by heterochromatin formation via histone H3 lysine 9 methylation [38,39]. Furthermore, histone hypoacetylation also contributes to its silencing [40]. *ade6*^*+*^ inserted in the silencing domain between the boundary and *mat2* (*L(BglII)*::*ade6*^*+*^) exhibits the typical variegated expression pattern [17]. Alternatively, a *cis*-DNA element–*cenH–*within the mating type region is sufficient to initiate heterochromatin formation at an ectopic site in the genome. *ade6*^*+*^ juxtaposed to *cenH* at an ectopic site (*ura4*::*cenH-ade6*^*+*^) also exhibits variegated expression [39].

**Fig 4:**
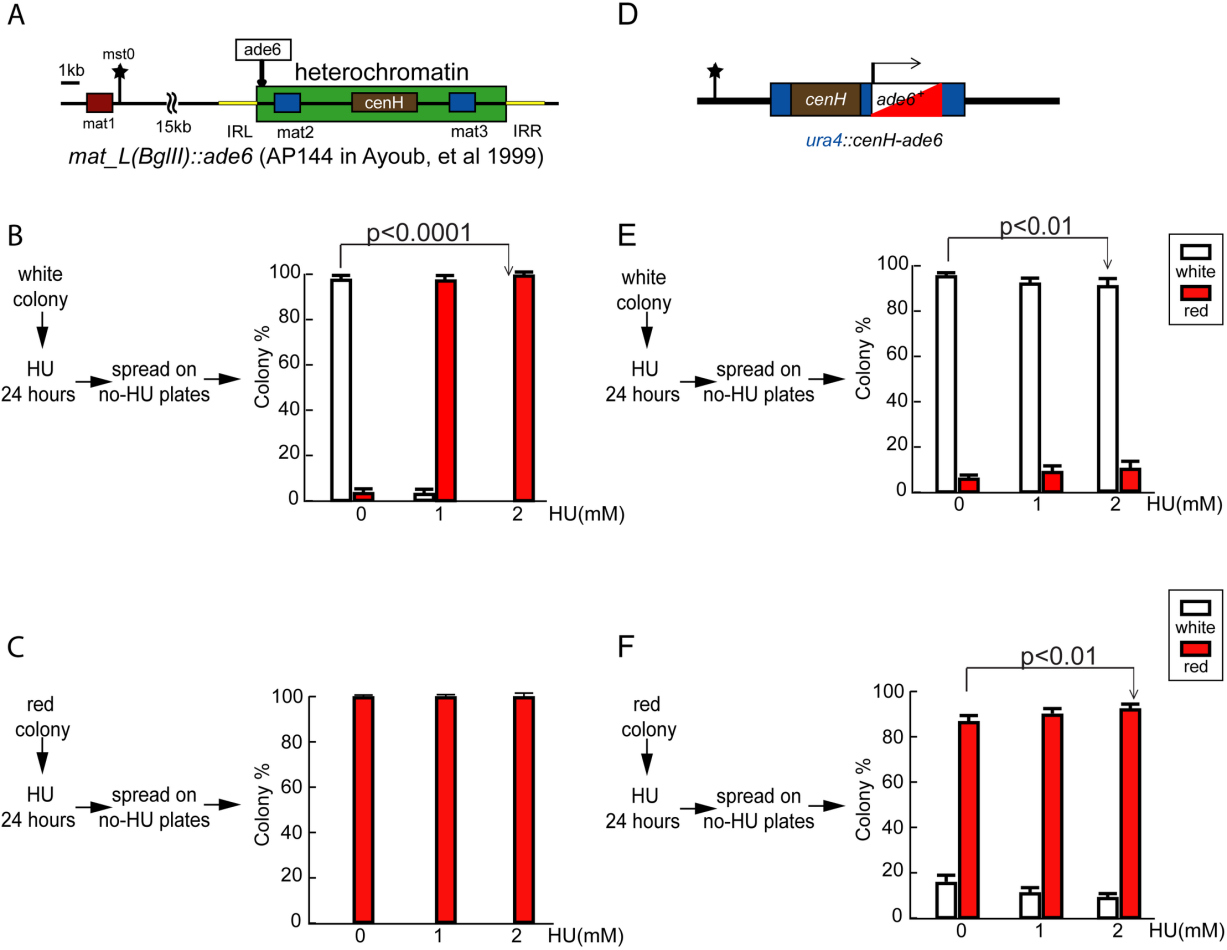
HU affects PEV at heterochromatin region. **(A)** HU affects PEV within the mating type locus. Diagram illustrates the location of *ade6*^+^ insertion at the mating type locus, between the centromere-proximal boundary and the *mat2-P* gene (strain AP144—*mat_L(BglII)*::*ade6*^+^). Dark green box indicates a normal silencing domain. **(D)** HU treatment affects PEV induced by *cenH* at an ectopic site. Diagram illustrates the genetic construct of the strain (LW75- *cenH- ade6*^*+*^). *cenH* and *ade6*^*+*^are inserted in the *ura4*^*+*^ locus, replacing the majority of the *ura4*^*+*^ ORF. **(B-C, E-F)** For each strain, cell suspensions of a predominantly white colony (B, E) and red colony (C, F) are treated with HU as labeled for 24 hours, washed and plated onto YE+4S solid media, and incubated at 25^°^C for 5 days. The red and white colonies are counted respectively, with 300–400 total/per plate. The error bars are 1 SD of percentages for four plates. *p* values are calculated by Fisher exact test.

We tested replication stress on PEV of *L(BglII)*::*ade6*^*+*^and *ura4*::*cenH-ade6*^*+*^, and found that HU increased *ade6*^+^ silencing in a dosage—dependent manner for both reporter constructs (Fig 4). Regardless cells originated from red or white colonies, when treated with HU, *ade6*^*+*^ silencing state was promptly established and persisted, resulting in red colonies with little or no white sectors. Between these two reporter constructs, *L(BglII)*::*ade6*^*+*^ exhibited a dramatic change, resulting in predominant or nearly all red colonies with HU treatment (Fig 4A–4C). In comparison, *ura4*::*cenH-ade6*^*+*^ exhibited a moderate but clear, unilateral increase in *ade6*^*+*^ silencing (Fig 4D–4F, the number of red colony is increased upon HU pulse treatment from 85% to 93% (start from a red colony), and the number of white colony is decreased from 97% to 90% (start from a white colony)). Such unilateral switching to silencing state is in sharp contrast to the observation in centromeric PEV using the same test, which resulted in colonies with enhanced bi-lateral switching. Upon re-plating to HU-absence media, white colonies re-emerged from *L(BglII)*::*ade6*^*+*^and *ura4*::*cenH-ade6*^*+*^ red colonies, suggesting that *ade6*^+^ silencing is due to epigenetic instead of genetic changes. To further confirm this notion, we examined the *ade6* gene in four of these red colonies by PCR amplification and DNA sequencing and found no mutation (S4 Fig).

Studies in the budding yeast have raised the concern that in certain experimental settings, reporter genes (URA3 and ADE2) may exhibit gene-specific transcription responses, rendering them unsuitable for characterizing the heterochromatin-induced silencing effects [41,42] (also see comments in [43]). To assess whether or not enhanced switching in *ade6*^*+*^ expression status in our experiments is reporter gene-specific, we tested the effect of replication stress on a native gene, *mat2-P*, at its endogenous locus. Mutations in specific genes (e.g., *clr1*, a zinc finger protein gene) partially compromise transcriptional silencing at the mating type region [37]. The leaky expression of *mat2-P* in stable M cells (*Mat1-Mmst0*—a genetic modification at *mat1* that locks the cell in the h- mating type) leads to haploid meiosis and sporulation, producing aneuploid, non-viable spores. Spore formation is detected by iodine vapor staining of the colonies or by microscopic examination of the cells. Importantly, iodine vapor staining reveals sectoring patterns, indicating that the silencing/leaky expression states of *mat2-P* are clonally inherited [44,45].

We examined *mat2-P* leaky expression in *clr1-*deletion *(clr1-D)* [46,47] colonies under replication stress conditions. The number of iodine staining patches and the intensity of staining diminished in an HU dose-dependent manner. Quantification of haploid meiosis (H.M. phenotype) within the colonies confirms a reduction in meiosis upon HU treatment (S5 Fig). *h*^*-*^*/h*^*+*^ diploid colonies under the same conditions are stained strongly by iodine vapor, suggesting that the low level of HU treatment used in this study does not inhibit meiosis or sporulation *per se*. These results suggest that low level HU treatment suppresses the leaky expression of *mat2-P* in *clr1-D* cells. To verify that this silencing effect is caused by heterochromatin on *mat2-P*, we further tested this notion by anti-H3K9me2 ChIP, and found heterochromatin is compromised in *clr1*-D strain (S5 Fig). HU treatment enhances H3K9me2 enrichment at *mat2-P*, demonstrating that heterochromatin underlies the gene silencing here. Thus, replication stress perturbs the inheritance of the expression states of a native gene similar to that of *ade6*^*+*^ reporter at the mating type region, arguing against the possibility of a gene-specific response to HU treatment.

In all, these results show that, unlike centromeric PEV in which the variation is enhanced, replication stress stimulates enhanced silencing unilaterally on two independent PEV systems mediated by H3K9me2/3, and the phenomenon is not reporter gene-specific.

### Replication stress facilitates spreading of heterochromatin at multiple loci of the genome

**Fig 5:**
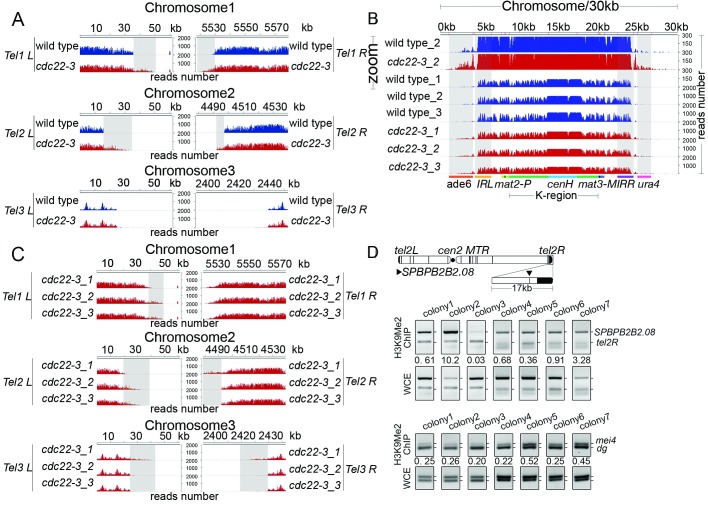
Replication stress promotes spreading of heterochromatin throughout the yeast genome. **(A)** H3K9me2 enrichment DNA reads in wild type and *cdc22-3* mutant cells are plotted over sub-telomeric region of all three chromosomes. Chromosome coordinates are indicated above the peaks. Scale bars on the right denote H3K9me2 enrichment DNA reads numbers normalized per one million reads. **(B)** H3K9me2 enrichment DNA reads in wild type and *cdc22-3* mutant cells were plotted over mating type locus. The location of the *ade6*^+^ gene (orange), the *IRL* repeats (yellow), *mat2-P* (light green), *cenH* (blue), the *IRR* (purple) repeats, and the *ura4*^+^ gene (pink) are indicated below the DNA peaks. Shaded areas highlight the *ade6*^+^, *IRL*, *IRR* and *ura4*^+^ genes. **(C)** H3K9me2 enrichment DNA reads in three biological *cdc22-3* mutant cells were plotted over sub-telomeric region of all three chromosomes. Chromosome coordinates are indicated above the peaks. Scale bars on the right denote H3K9me2 enrichment DNA reads numbers normalized per one million reads. **(D)** Multiplex PCR is performed on immune-precipitated DNA from 7 independent yeast colonies of *cdc22-3* mutant to amplify a fragment from the gene (*SPBPB2B2*.*08*) nearby sub-telomere right region in chromosome II, as well as a fragment from *tel2R* locus. For the normalization control, PCR is performed in parallel from non-immunoprecipitated DNA (WCE panel). The numbers shown are calculated by the gray value of PCR product from ChIP samples divided by the gray value of that from WCE samples.

Net enhanced silencing of heterochromatin-associated PEV by replication stress at multiple loci in the genome may be explained by a possible mechanism that the heterochromatin domains are expanded. Consistent with this hypothesis, Singh and Klar previously have shown that *cdc22-3* causes heterochromatin silencing and H3K9 methylation spreading at the silent *mat* locus[25]. To test whether such effect is broadly seen throughout the genome, we compared heterochromatin distribution on whole genome-wide in wild type cells with or without HU treatment and *cdc22-3* mutant cells by ChIP-Seq. In specific, chromatin immunoprecipitation was performed in these cells using an antibody against histone H3 dimethylated at lysine 9 (H3K9me2). And the immunoprecipitated DNA was then subjected to high throughput sequencing to determine the specific location and relative abundance of H3K9me2 throughout the genome. The result showed there is no appreciable difference in wild type cells with or without HU treatment. However, comparing between wild type and *cdc22-3* mutant cells, significant difference can be seen at mating type locus and sub-telomeric regions (Fig 5A and 5B. Please see below for more in-detailed analysis of the ChIP-seq results). We are unclear why short-term HU treatment in wild type cells didn’t cause significant changes in heterochromatin distribution whereas genetic perturbation of *cdc22-3* mutation did. We speculate that this may be because cells within a culture treated with HU temporarily are highly heterogeneous in terms of epigenome perturbation. Any changes in epigenome at the specific locus in a small percentage of cells may not be detected readily by ChIP-seq.

Throughout the fission yeast genome, two types of heterochromatin have been found–constitutive heterochromatin domains and facultative heterochromatin islands [48,49]. The former are strong, persistent heterochromatin domains, including peri-centromeric, sub-telomeric regions and the mating-type locus. The latter encompasses ~30 loci, the majority of which are meiosis genes [49].

In wild type cells, we have detected H3K9me2 mainly at the constitutive heterochromatin domains, identical to previous reports [49] (S6 Fig). We have also detected a number of heterochromatic islands with relatively low levels of H3K9me2, most in agreement with previous reports. A few heterochromatic islands are different from those reported in recent studies [49,50]. The discrepancies might be caused by the difference in sensitivity of the experimental tools (ChIP-chip vs ChIP-Seq), or different data processing methods.

In *cdc22-3* mutant, we detected alterations in some of the heterochromatin domains in comparison to wild type (Fig 5A and 5B). Specifically, H3K9me2 enrichment at the sub-telomeric regions is expanded by 5-20kb. And the heterochromatin at the mating type locus spreads beyond the normal boundaries to the neighboring genes, just as described in Singh and Klar’s work[25].

To confirm the heterochromatin expansion, we tested the effect of HU on mating type locus using reporter genes inserted outside the wild type mating type boundaries [25]. Consistently, we have observed enhanced reporter gene silencing under HU treatment (S7 Fig, *ura4* gene silencing cells exhibit resistance at 5-FOA plate), similar to what was previously reported [25].

Expansion of the heterochromatin domains is specific to the regions described above. No change was detected in the peri-centromeric regions of any chromosomes. This suggests that alteration in heterochromatin expansion induced by replication stress is locus-specific.

Noticeably, we observed that the detected alterations of heterochromatin in mutant cells were variable among biological repeat samples (Fig 5C), whereas the positions of heterochromatin domains are highly consistent among wild type biological repeats (S6 Fig). In mutant Sample 1 (*cdc22-3_1*), the heterochromatin of *Tel1 L* and *Tel2 L* was shorter than the parallel biological repeats (*cdc22-3_2* and *cdc22-3_3*), whereas the heterochromatin of *Tel2 R*, *Tel3 L*, and *Tel3 R* was longer than the parallel biological repeats (Fig 5C). The variation among the samples is unlikely due to technical reasons, as such variation is seen only in the sub-telomeric regions, while other regions of the genome are highly consistent.

Such sample-specific chromatin structure changes may indicate they are sporadic events among genetically identical cells/cultures and are epigenetically inheritable. To validate this notion, we examined the variation of heterochromatin spreading using ChIP-PCR among seven independent yeast colonies derived from the same ancestor cells (Fig 5D). Using primers to amplify a fragment nearby *tel2R* (*SPBPB2B2*.*08*, with low level of H3K9me2 enrichment in wild type cells) along with primers set that amplified a fragment of *tel2R* (with high level of H3K9me2 enrichment in wild type cells) as a control, we found that three to ten folds enrichment of *SPBPB2B2*.*08* gene fragment among different samples, whereas constant levels of *mei4* and *dg* are detected throughout all the colonies (Fig 5D). Thus, varied expansion of heterochromatin at sub-telomeric regions from genetically identical cells (*cdc22-3* mutation in this case) occurs presumably randomly and once established, is relatively stable.

### Replication stress affects PEV and development in *D*. *melanogaster* and *C*. *elegans*, respectively

Chromatin organization and its inheritance through the cell linage are crucial for cell fate specification and cell identity maintenance during development in metazoans. We postulated that replication stress might induce chromatin change and alter gene expression and therefore, perturb the development process.

To test this notion, we first ask whether HU treatment may affect the inheritance pattern of epigenetic marks in *Drosophila*, using PEV of the *white* gene expression as the readout. The X chromosome inversion *In(1)w*^m4^ brings the *white* locus near to the heterochromatin. Spreading of the silent chromatin marks causes a highly variable mosaic pattern in eye coloration.

**Fig 6:**
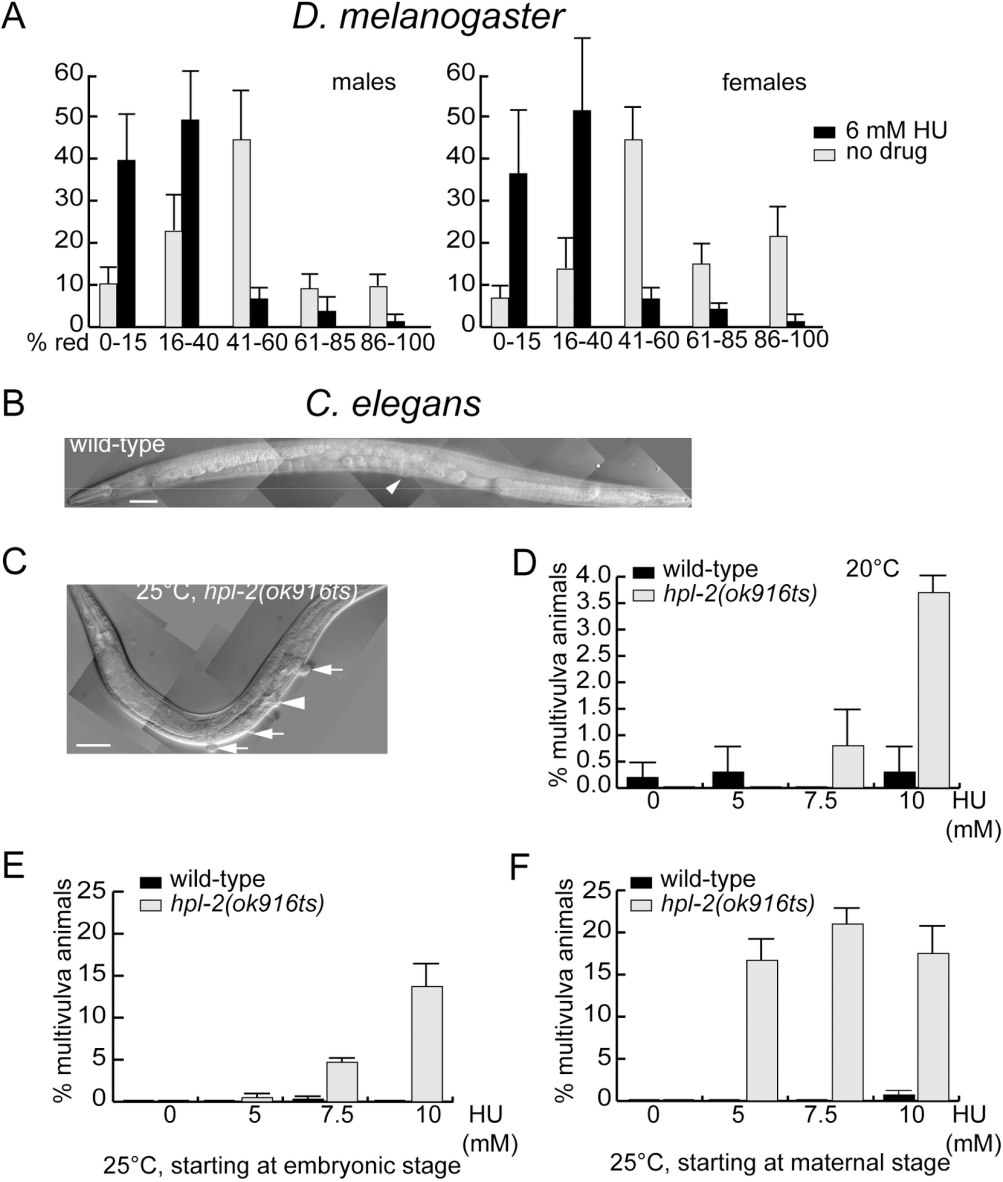
Replication stress affects white gene PEV in *D*.*melanogaster* and vulva development in *C*.*elegans*. **(A)** HU treatment increases the likelihood of *In(1)wm4* silencing in flies. Male or female flies grown without drug (gray bars) or 6 mM HU (black bars) were separated into five bins based on the degree of eye pigmentation. Bar height is the percentage of the population in that bin for all pooled trials. The error bars are 1 SD of percentages for four or five independent trials. Total flies assayed: > 500. (B-F) HU treatment induces a *SynMuv* phenotype in an *hpl-2* mutant worm. **(B)** Montage of DIC images of a wild-type with one normal valva (arrowhead) and **(C)** an hpl-2 mutant adult raised on 10mM HU plate, with three vulva-like structures (arrows). Scale bars are 50 mms. **(D-F)** Synchronized L1 larvae were placed on NGM plates containing HU as labeled, the multivulva phenotype was scored after worms reached adulthood. Each data point is represented as mean ± SD. In (D), the animals for scoring and their mothers were raised at 20°C; in (E), mothers were raised at 20°C, and the animals for scoring were raised at 25°C, start at the embryonic stage; in (F), the animals for scoring and their mothers were all raised at 25°C.

Fly larvae are treated with HU and the eye coloration patterns are quantified in adult flies. Flies are sorted into five bins based on the degree of pigmentation (Fig 6A). Bin 1 contains flies with nearly pure white eyes (silenced *w* locus) with only a few pigmented omatidia. The eyes of flies in bin 3 contain roughly equal sectors of red and white tissue. Bin 5 flies have eyes that were solid red (fully active *w* locus). When parallel cultures of flies are fed with 6 mM HU throughout larval development, roughly half the animals that form pupae die at that stage, but those that reach adulthood are placed in the same five bins based on eye pigmentation. We have found a strong shift in the distribution towards whiter eyes (Kolmogorov-Smirnov comparison *p*< 0.001), indicating greater probability that the *w* locus is silenced (Fig 6A). The same result is obtained for females carrying two copies of the *w*^m4^ locus and males with only one. This suggests that replication fork pausing in *Drosophila* reduces the fidelity in chromatin organization duplication.

We also test whether HU treatment affects cell fate specification during vulva development in the nematode *C*. *elegans*. A number of genes implicated in vulva development encode chromatin factors, including heterochromatin binding protein (*hpl-2*), histone methyl-transferase (*met-2*) and nucleosome remodeling protein (*ssl-1*), supporting a prominent role of chromatin structure in vulva development [51]. These genes and others constitute three redundant genetic pathways–SynMuv (Synthetic Multi-vulva) A, B and C–that control cell fate during vulva development. Simultaneous inactivation of two pathways results in abnormal induction of vulva cell fate and creates multiple vulva-like structures in contrast to the normal, single vulva [51].

We examined whether HU treatment would enhance the synthetic multi-vulva (SynMuv) phenotype of *hpl-2* (Fig 6B and 6C). The *hpl-2 (ok916ts)* allele, a truncation of *hpl-2* encoding only the N-terminal one third of HPL-2, displays a SynMuv Phenotype at 25°C (restrictive temperature) but not 20°C (permissive temperature) together with a SynMuv A mutation [52]. No Muv phenotype is observed in *hpl-2* single mutants at either temperature ([52] and Fig 6D–6F). Synchronized L1 larvae of wild type and *hpl-2* worms are placed on plates with HU, and the SynMuv phenotype is scored when worms reach adulthood. None of the HU concentrations we use stopped worm development at either 20°C or 25°C (Fig 6D–6F), indicating that these dosages do not completely block DNA replication or cell cycle progression. When worms are raised at 20°C and treated with HU, only the highest HU dosage (10 mM) results in a significant percentage of SynMuv animals in *hpl-2* mutants but not in wild type (Fig 6D). When samples are raised at 25°C, a much more dramatic, HU dosage-dependent increase in the percentage of SynMuv animals is observed (Fig 6E). The most obvious induction of the SynMuv phenotype is observed from 25°C-raised *hpl-2* mutant animals that are descendants of mothers that have been raised at 25°C. The HU-induced SynMuv phenotype associates with *hpl-2* mutation indicates that chromatin duplication and/or remodeling is mis-regulated by HU treatment in *C*. *elegans*.

In conclusions, these results demonstrate that reduced fidelity in chromatin duplication induced by replication stress is an evolutionarily conserved phenomenon and that, in a suitable cellular context, it causes defects in cell fate specification and development.

## Discussion

Various external or intracellular factors such as metabolic stresses, genotoxic insults, deregulation of replication and oncogene activation could induce replication stress, which is a major source of genome instability and a hallmark of most cancer types.

In addition to genetic instability, changes in chromatin structure have also been associated with replication stress[9,10,25], suggesting that replication stress may also affect epigenetic inheritance. Here, we present a series of *in vivo* evidence in three organisms that replication stress disturbs chromatin duplication, causing epigenetic instability. Our results highlight the importance of coordination between replication and nucleosome assembly to ensure accurate chromatin duplication.

### Replication stress broadly induces epigenetic instability

Previous study shows that failure to maintain processive DNA replication at G4 DNA in REV1-deficient cells leads to uncoupling of DNA synthesis from histone recycling, resulting in a local tract of chromatin lacking the parental epigenetic marks [53]. Chromatin alterations can arise as a consequence of perturbed histone dynamics in response to replication stress, which may facilitate stochastic epigenetic silencing by laying down repressive histone marks at sites of fork stalling[54]. Our in-detailed study in the fission yeast *S*.*pombe* expands this notion. Using reporter gene expression states as the readout, we demonstrated that replication stress affects the precision of inheritance of an epigenetic trait on chromatin—Cnp1 occupancy in the centromere. We further demonstrated that replication stress affects heterochromatin distribution at multiple loci in the genome. Together, these results show that replication stress broadly affects chromatin-mediated epigenetic inheritance. Our results in the fruit fly and worm studies further illustrated the broadness and the evolutionary conservation of the phenomenon.

Finding that replication stress affects epigenetic stability is potentially important for elucidating the mechanisms of its functional role, for instance, in tumorigenesis. In cancer development induced by aberrant activation of the Rb-E2F pathway, the primary cause of replication stress is the reduced nucleotide pool in cells [55], a condition similar to the HU-induced or *cdc22-3* mutation-dependent replication stress tested here and in a previous study[25]. Our current work and others [9,10,25,26,56] support the model that, in addition to its well-established role in genetic instability, replication stress may also lead to epigenetic instability, providing an additional mechanism for cancer initiation and progression. Furthermore, our analysis of worm vulva’ development showcases the impact of replication stress in perturbing embryonic development via epigenetic instability.

### Mechanisms underlying epigenetic instability

**Fig 7:**
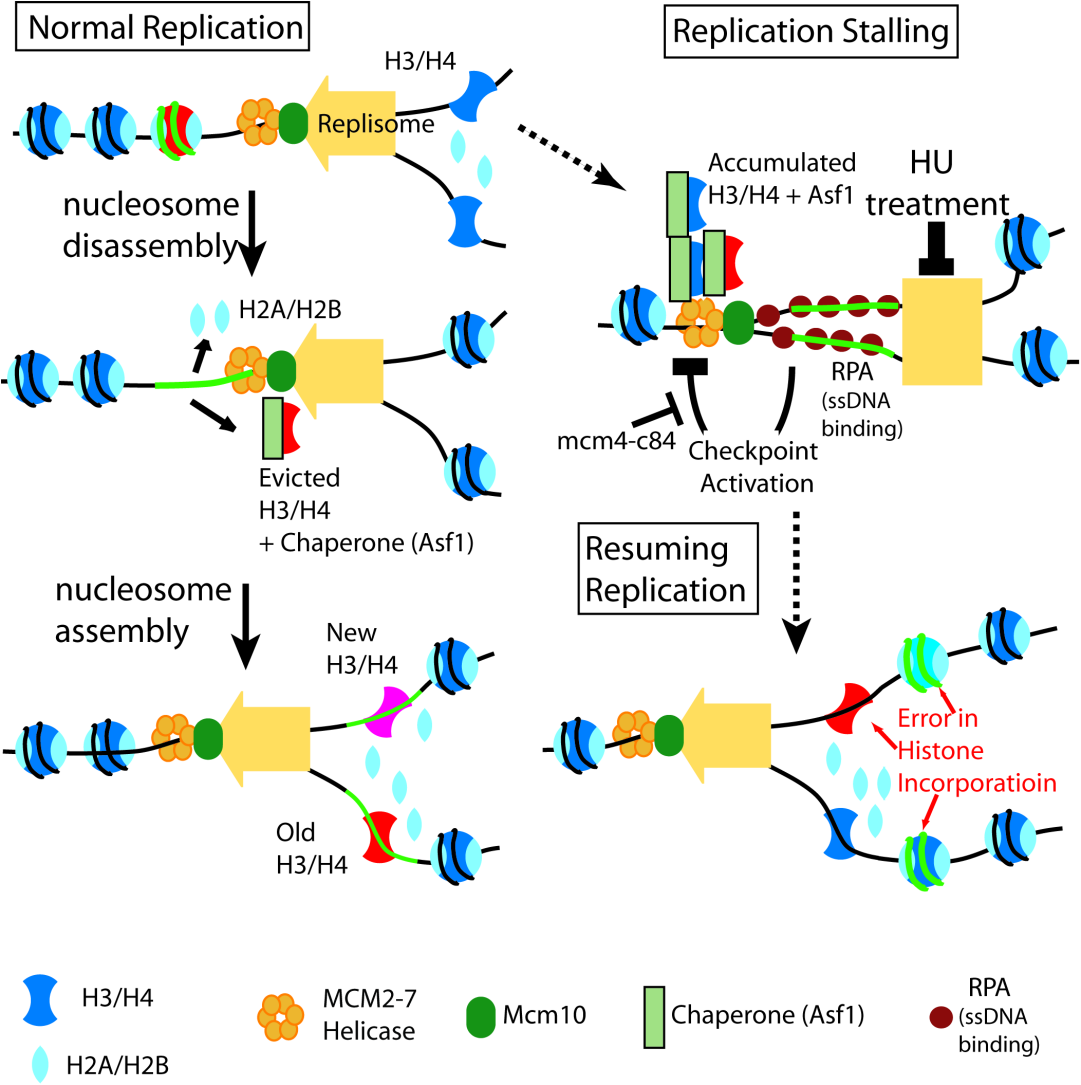
Replication stalling affects RC nucleosome assembly. Left: normal RC nucleosome assembly. Individual nucleosomes are distinguished by colors. Accurate duplication of chromatin is exemplified by the “red” nucleosomes maintaining association with the “green” DNA segment after replication. Right: during replication stalling (HU), helicase continues unwinding DNA, accumulating evicted parental histones and forming ssDNA, which cause errors in parental histone recycling after replication resumes. Mutant such as *mcm4-84c*, that causes excessive formation of ssDNA, aggravates the defect in nucleosome inheritance.

Based on the biochemical studies in human cells, Jasencakova *et al* has proposed the model that replication stress may promote chromatin structure change by perturbing parental histone recycling in replication-coupled nucleosome assembly [26]. Their results have illustrated that with replication stalling, a complex comprised of the histone chaperone Asf1, histone H3-H4 dimer and the MCM helicase accumulates in nucleus, with the histones carrying the parental molecule signatures. Upon recovery from replication stalling, the accumulated histones are recycled and incorporated in the replicated chromatin [26]. It is possible that the excessive amount of ssDNA binding with RPA, formed in the presence of replication stresses, may bind the evicted parental histones and new histone H3-H4. This speculation is supporting by a recent study in budding yeast, demonstrating that during normal replication, ssDNA binding protein RPA binds histone H3-H4 and multiple histone H3-H4 chaperones and promotes the deposition of H3-H4 onto adjacent dsDNA[56]. If validated under the conditions of replication, this would provide a molecular mechanism for how replication stress may affect the chromatin epigenetic inheritance stability, causing broad alteration in the nucleosomal organization pattern [57,58] (Fig 7). At this stage, we cannot rule out the possibility of global perturbations to the physiological state of the cells due to HU treatment and/or genetic mutations may underscore the epigenetic stability perturbation. An alternative model for the above observation may be that the epigenetic instability induced by replication stress is a consequence of changes in cellular homeostasis and physiological state, for example, altered histone protein supplying due to S phase delay/arrest. However, the fact that checkpoint inactivation, which prevents the delay/arrest of S phase but exacerbates ssDNA formation, enhances the epigenetic instability in centromere (Fig 2) argues against this notion.

Studies in chick DT40 cells lacking the translesion synthesis polymerase REV1 showed that non-Watson-Crick G-quadruplex (G4) DNA causes persistent replication stalling, leaving gaps in DNA that are filled in a post-replication manner [59]. Such severe perturbation in replication leads to loss of local histone modification signature and changes in gene expression state. In this case, it is postulated that a failure to recycle the parental histones at all during post-replicative filling-in of the gaps [53], providing an alternative mechanism for epigenetic instability induced by DNA damage repair. Interestingly, in fission yeast *mcm4-M68* cells at the semi-permissive condition, although there was no excessive ssDNA formation in S phase, strong Ssb2-GFP signal accumulation was detected in G2 phase (S3 Fig). This is consistent with the previous finding that *mcm4-M68* cells delay cell cycle progression in G2 phase and require the Chk1-dependent DNA damage checkpoint for survival [33]. Nonetheless, these cells exhibited near-wild type levels of centromeric PEV, indicating that in contrast to REV1-deficient chick DT40 cells, here, post-replication repair of DNA damage does not induce epigenetic instability in centromere. The reason underlying this discrepancy is unclear. One possible explanation is that DNA damage due to failed replication initiation could be specifically localized at the sites of replication origins [33], thus, its repair may only affect chromatin structure locally (such as what was observed in REV1-deficient chick DT40 cells [53], [58]). It is also noteworthy that neither the wild type cells nor the *mcm4-M68* mutant cells show an accumulation of ssDNA at 29°C as well as 25°C (Fig 3 and S2 Fig). However, the switch frequency is increased appreciably (although quite low) in 29 degree compared with that in 25 degree in wild type cells. This may be because the current method of Ssb2-GFP foci measurement is not as sensitive (and accurate) as needed to detect a possible minor ssDNA accumulation. Or, the high temperature may affect the epigenetic inheritance stability via a mechanism other than ssDNA accumulation.

The above mechanistic model highlights the contribution by impaired parental histone recycling to epigenetic instability. It is conceivable that such effect is broadly applicable throughout the epigenome. Although, this does not exclude the possible contribution of defects in other steps of RC nucleosome assembly in association with replication stalling, such as the maturation of newly assembled nucleosomes in which new histones obtain proper post-translational modification marks.

Specifically for heterochromatin organization in fission yeast genome, in addition to covalent modification signature carried by the parental histone molecules (H3K9me2), multiple mechanisms, such as RNAi, specific DNA element-binding proteins and HP1-mediated local spreading of histone H3 modification, are at play to establish/maintain heterochromatin in a locus specific manner[9]. These mechanisms may also be affected by replication stresses. For example, Singh and Klar found that mutations in *cdc22* cause heterochromatin spreading beyond the silent *mat* locus[25], may via an indirect mechanism of increased recruitment of Swi6 and Clr4 by the stress-induced transcription factors Atf1/Pcr1. Consistently, we found that Atf1 mRNA expression level is increased significantly in *cdc22-3* mutant cells (S8A Fig). Furthermore, ChIP analysis indicates that levels of Swi6 at the heterochromatin region and on the reporter genes outside the mating type boundaries (S8D Fig) are increased in *cdcd22-3* mutant cells, supporting the notion that the enhanced spreading of heterochromatin at mating type locus and sub-telomeric region under replication stress may also via an indirect mechanism of increased Atf1-mediated recruitment of Swi6. Alternatively, removal of the jmjC protein Epe1 that antagonizes heterochromatin spreading beyond its normal borders by Cul4-Ddb1^Cdt2^, may also lead to expansion of the heterochromatin domain[60–62]. However, we found the levels of Epe1 are increased at all of the investigated heterochromatin sites and decreased at the *ade6* gene outside the mating type boundary when under replication stress (S8E Fig). The reason underlying this discrepancy is unclear. The increased binding of Epe1 in heterochromatin region might be a result from the up-regulated bindings of Swi6 when under replication stress. Considering that the process of Pol II-dependent transcription through heterochromatin and siRNA formation are restricted to S phase [63,64]and are also affected by Epe1[60], it is possible that in prolonged S phase with replication stress, increased binding of Epe1 would stimulate the binding of Pol II to heterochromatin and thus the formation of siRNAs during S phase; subsequent removal of Epe1 by Cul4-Ddb1^Cdt2^ would then allow assembly of heterochromatin. Further experiments are needed to test this in future.

Comparing between *mst0 clr1D* cells with or without HU treatment, we also detected a slight increase in *atf1* mRNA expression. However, no significant difference was found in terms of Swi6-9myc or Epe1-9myc association at the mating type locus. More evidence is needed to support that HU promote heterochromatin silencing in *clr1D* cells via Atf1-mediated recruitment of Swi6 as well as reduced level of Epe1 at mating type locus boundary.

To some extent, these results suggest that the direct impact of replication stress on epigenetic inheritance stability may vary, at least for heterochromatin, at various loci within the genome. Further supporting this notion, we have found sample-specific chromatin structure changes at sub-telomeric regions in three biological mutants with good correlations (Fig 5C and 5D), which suggest they are sporadic events and are epigenetically inheritable. Thus, while replication stress can alter epigenetic stability and chromatin structure at multiple loci (and perhaps throughout the whole genome), so far, there lacks a common pattern on how epigenetic stability and chromatin structure may be changed. Such change may likely vary depending on the local chromatin context, the biochemical nature of the epigenetic marker. And in some cases (such as the heterochromatin distribution at the sub-telomeric regions), the changes appear to be sporadic and variable among individual cells. Perhaps as an indication of additional levels of complications in the mode of heterochromatin change due to replication perturbation, earlier studies demonstrate that gene silencing due to heterochromatin decreased in certain replication mutations (*swi7* and *mcl1*, defective in DNA polymerase alpha [10,65]), in contrast to a unilateral increase as demonstrated in these studies. It remains to be tested whether replication perturbation due to mutations in *mcl1* and *swi7* causes accumulation of ssDNA. Clearly, further studies are needed to better understand how the replication stresses affect epigenetic stability of the whole epigenome in general as well as distinct domains of the chromatin in a context-specific manner.

## Materials and methods

### Yeast strains, medias, and the culture conditions

*S*.*pombe* strains used in this study are listed in S1 Table. Yeast strains are constructed by either random spore method or by tetra analysis. Yeast cells are grown on YE+5S medium (add with 5 supplements including histidine, uracil, lysine, leucine and adenine) or YE+4S medium (add with histidine, uracil, lysine and leucine, except adenine is provided by yeast extract, it is sufficient to provide the growth for cells with *ade6*^*+*^ transgene inserted in the centromere or mating type locus and for colony color differentiation). Solid malt extract (ME) medium or synthetic medium without nitrogen (EMM-N) is used for mating and sporulation. β-estradiol (Sigma) at 100nM in YES is used for growth of *psf1-HBD* mutants, with β-estradiol at 0.1nM in YES for *psf1-HBD* depletion.

### Pedigree analysis

Pedigree analysis is performed as described [16] with modifications to suit specific experimental conditions. To track the cell lineage for each three-generation family, cell suspension is spread in a line near the top of a thin YE+4S plate. Eight parallel lines are drawn below the line of cell spreading on the back of the plate using a marker pen. Using a tetrad dissection microscope (Nikon), one cell is picked and moved onto the first line and the plate is incubated at 25^°^C, allowing the cell divide into two cells. One of the daughter cells is then moved onto the fifth lines. In the ensuing generations, always leave one daughter at the original position and move the other to a designated position. After three generations, eight progeny cells are placed at specific positions, and allowed to grow into colonies by incubation at 25^°^C for 5 days.

To test whether HU treatment affects the switch frequency, a “hybrid” plate with a HU-free zone and a HU-containing zone is used. Cell suspension is spread in a line near the top of HU containing zone. One cell is picked and moved to the nearby location, allowing the cell divide into two cells. Then separate these two cells, waiting their division. Keep doing this step until the cell finished third generation. Move these eight progeny cells into HU-free zone at the designated position, incubate at 25^°^C for 5 days.

To test the effect of partial inactivation of *mcm4-M68* at 29^°^C, three-generation tracking is performed at 29^°^C. The plates are then incubated at 25^°^C for 5 days before scoring.

### Microscopy

Logarithmic growth cells are treated with specified concentrations of HU for 6 hours, and the Ssb2-GFP signals are measured under the microscope. Photomicrographs are obtained using a Delta vision core and personal DV. First, use the “Deconvolve” function to deconvolve all z-stacks. Then, use the “Quick projection” function to combine all z-stacks, choosing the “maximum intensity”. In “Edit Polygon” function, use the freehand polygon to draw a circle around the bright dot, reading the raw number. And put this same size of circle at the middle of cells, reading the number as background. Ssb2-GFP signal of this dot should be the subtraction of these two numbers. The total Ssb2-GFP signals in each nucleus equal the sum of all the detectable dots within the nucleus. Image processing and analysis are carried out using Soft WORX 3.2.2 software and Adobe Photoshop. Ssb2-GFP signals in wild type cells are normalized as 1X.

### Iodine staining

Individual colonies grown on sporulation (EMM-N) medium for 5 days at 30^°^C are exposed to iodine vapors to stain a starch-like compound produced by sporulating cells. In mating-type switching-defective strains, such as *mat1-Msmt0*, the intensity of dark brown/black staining indicates the level of sporulation.

### Chromatin immunoprecipitation (ChIP) analysis and high throughput sequencing

ChIP analyses with H3K9me2 antibody (Abcam, ab1220) and anti-myc antibody (Abcam, ab9132) are performed as described previously [16,66]. Log-phase cells of wild type and *cdc22-3* mutant are grown at 30^°^C in YE5S media, as described in Singh and Klar’s work [25]. The 10^9^ cells were harvested and digested by Zymolyase 20T with final concentration of 0.25mg/ml for almost one hour at 37^°^C. For H3K9me2 ChIP analysis, the chromatin was pre-warmed to 37^°^C for 5min, followed by the addition of micrococcal nuclease (Thermo EN0181) to a final concentration of 240U/ml for 30min. The digestion reactions were incubated at 37^°^C with gentle rolling, and were immediately stopped by the addition of EDTA to a final concentration of 2mM. After centrifugation, the ChIP reaction was performed as before. For anti-myc ChIP analysis, 2*10^8^ cells were harvested. And the chromatin was cross-linked by 1% final formaldehyde and stopped by adding 125mM final glycine. The chromatin was sheared by sonication with 30S ON and 30S OFF for 30 cycles.

Multiplexed libraries are prepared at the same time using the library preparation kit (KK8301) from kapa biosystems, and the barcode adapters from Life Technologies (014D01-14). All the libraries are sequenced on Ion torrent_PGM (200bp sequencing kit) with one 318 chip, and approximately 0.5–0.8 million aligned reads per sample are taken.

Portions of immunoprecipitated DNAs and whole cell extract from seven independent colonies of *cdc22-3* mutant are used as the PCR template. The primers are set to amplify a fragment from telomere nearby locus (*SPBPB2B2*.*08*) along with that amplified a product from sub-telomere region (*tel2R*). Primers amplify meiosis gene *mei4* as well as *dg* fragment of centromere are set as controls. The locations of the primer sets are shown in Fig 5D. The numbers shown in Fig 5D are calculated by the gray value of PCR product from ChIP samples divided by the gray value of that from WCE samples.

### Quantitative real-time PCR (qPCR)

All real-time PCR were done with a Bio-Rad CFX96 Touch. All samples were run in triplicate to ensure accuracy of the data, and their average was calculated. PCR of 45 cycles was done using SYBR Green qPCR kit (Bio-Rad172-5120). Primers were used at 0.3uM for each experiment. The PCR product length was about 120bp. ChIP analysis was similarly performed by quantifying the amount of DNA in ChIP samples without antibodies (beads only), using the same apparatus and reagents. ChIP/WCE was determined by calculating their *Ct* value difference, followed by subtracting the *Ct* value difference between beads only and WCE. The primer sequences for qPCR are available upon request.

### *Drosophila* HU treatment and eye color characterization

Instant Drosophila food (Carolina Biological) was prepared with either water or water containing 2, 5, 7, or 10 mM hydroxyurea and supplemented with a few grains of dry yeast on the surface. *In(1) w*^m4^ parents laid eggs for three days and were moved to new food vials. Few progeny were recovered from the 10 mM HU vials, but approximately half of the third instar larvae growing on 7 mM HU successfully completed pupation allowing adult eye pigmentation to be scored. The adults were sorted by sex and then by the degree of eye pigmentation (Bin 1: pure white-~15% pigmented, Bin 2: ~15–40%, Bin 3: ~40–60%, Bin 4: ~60–85%, Bin 5: 85%- solid red). In most cases both eyes of a single fly had comparable pigmentation, but when there was an obvious difference, the fly was placed in the bin for the darker eye. The experiment was performed in four trials for the–HU control (total 726 adults) and five times for the 7 mM HU assay (total 527 adults).

### *C*.*elegans* HU treatment and SynMuv phenotype characterization

HU were added to NGM plates at indicated concentrations. Before *E*. *coli* (worm food) was seeded on the NGM plates, HU was also added to the *E*. *coli* suspension so that the HU concentration in the *E*. *coli* suspension matches that in NGM plates. Gravid mothers that were raised at either 20°C or 25°C were bleached and eggs collected on NGM plates that were without *E*. *coli*. After 24 hours incubation at 20°C or 25°C, synchronized L1 larvae were placed on NGM plates containing different concentrations of HU and incubated at 20°C or 25°C. Adults that were 24–48 hours post-L4 stages were scored for the SynMuv phenotype.

### Statistical analysis

Data are presented as the mean±SD. Statistical analysis was made for multiple comparisons using analysis of variance and Student’s *t* test or Fisher exact test. A *p* value <0.05 was considered to be statistically significant.

### Data access

Reads are mapped to *S*. *pombe* ASM294v2 assembly using “bwa” with default parameters, and only the uniquely mapped reads are obtained for further analysis. Nucleosome position and occupancy are calculated by DANPOS [67], which normalize data by random sample and adjust clonal signal to 1 read. Reads are adjusted to 73 bases with 5’ fixed when occupancy is counted. IGV is chosen for data visualization, and the annotation file (which is updated to May 2015) for *S*. *pombe* ASM294v2 assembly is downloaded from website (http://www.pombase.org).

### Accession numbers

All ChIP-Seq data have been submitted to GEO Datasets under accession numbers [GSE89816].

## Supporting information

S1 Fig Replication perturbation changes colony morphology in different mutants. (A) *cds1-D* (LW21) and *mcm4-84c* (CY344) colonies exhibit enhanced sectoring at the presence of HU. Cells of the mutant strains and wild type (XL762) are planted at the density of one cell per cm^2^ on YE+4S plate containing HU as labeled, and incubated at 25^°^C for eight days. (B) High degrees of sectoring on the colonies of the replication mutants. Wild type, *ctf8-D* and *ssb3-D* cells are planted on YE+4S plates, and *psf1-HBD* cells on YE+4S+100nM β-estradiol and YE+4S+0.1nM β-estradiol, respectively, incubated at 25^°^C for eight days. Wild type and *mcm4-M68* cells incubated at 29 ^o^C for six days. One representative colony for each was shown. Scale bar is 2mm.
(TIF)

S2 Fig The protein levels of Ssb2-GFP are unchanged in mutant cells with or without HU treatment. The protein levels of Ssb2-GFP are examined by western blot analysis, using ribosome protein S6 as a negative control. The gray value of each band is measured, and the ratios of GFP/S6 of the indicated strains were normalized to the value of GFP/S6 in wild type without HU treatment.
(TIF)

S3 Fig The biological activity of MCM helicase is compromised at *mcm4-M68* mutant at semi-permissive temperature. (A) *mcm4-M68* (LW22) mutant cells show elongated cell shape compare with wild type cells at 29°C. Both mutant and wild type cells are grown at 25°C over night, then shift to 29°C for 6h. Cells are harvested and fixed with methanol. After washing with PBS, cells are stained with Hoechst dye for microscopic examining of the nuclear morphology. Scale bar is 10μm. (B) *mcm4-M68* mutant cells exhibit mild reduced survival rate at 29°C. Cells are grown at 25°C over night. Cell suspensions of both strains are plated onto YE+5S solid media with 400 colonies per plate, and incubated at 25°C and 29°C separately. The number of survival colonies was counted. (C) Increased levels of Ssb2-GFP signal in the mutant G2 phase cells. Wild type, *mcm4-M68* cells are grown in the liquid YE+5S media at 25°C over night, then shift to 29°C for 6h. Ssb2-GFP signal is measured as in Fig 2A. Representative G2 phase cell images are shown in the right panels. Scale bar is 3μm.
(TIF)

S4 Fig DNA sequencing alignment of *ade6*^+^ gene in the strains of *L(BglII)*::*ade6*^*+*^and *ura4*::*cenH-ade6*^*+*^. Four red colonies from *L(BglII)*::*ade6*^*+*^and *ura4*::*cenH-ade6*^*+*^ strains were picked and the *ade6*^+^ reporter was PCR amplified and subjected to sequencing. No mutation was found in *ade6*^+^ in these colonies. DNA sequence alignment for one of the four sequencing results is shown. Query: DNA sequence of *ade6*^+^ PCR product from red colonies. Subject: DNA sequence of *ade6*^+^ gene downloaded from website (http://www.pombase.org).
(TIF)

S5 Fig Replication stress affects the inheritance of the endogenous *mat2-P* gene expression status at its native locus. (A) The diagram illustrates the genetic construct of the mating type locus. Light green box indicates a compromised silencing domain. (B) HU treatment restores the silencing of *mat2-P* at the mating type locus in *clr1*-*D* strain. *mat1-msmt0 clr1*-*D* haploid cells (LW63) and *h*^*-*^*/h*^*+*^ wild type diploid cells (LW52) are plated on sporulation medium (EMM-N) containing HU as labeled, incubated at 30^°^C for five days, stained with iodine vapor. Two colonies each are shown. Cell suspensions of the colonies are then stained with Hoechst dye for microscopic examining of the nuclear morphology. The percentage of *clr1*-*D* cells showing a haploid meiosis phenotype (H.M.) is quantified in five independent colonies with >100 cells scored in each. (C) HU treatment promotes heterochromatin in *clr1*-D strain. *mat1-msmt0 clr1*-*D* haploid cells (LW63) were cultured in EMM5S liquid media until *OD*_*600*_ reached 0.1, then shifted to EMM-N liquid media with or without 2mM HU for 17 hours. Cells were harvested and immuno-precipitated with anti-H3K9me2 antibody. Heterochromatin enrichment was examined by ChIP using quantitative real-time PCR. Recovery ratios of immuno-precipitated DNA to total DNA at the indicated loci were normalized to the value of tubulin gene. Data are mean ±s.d. (n = 3).
(TIF)

S6 Fig Mapping H3K9me2 reveals heterochromatin islands. (A) Relative fold enrichment of dimethylated H3K9 (H3K9me2), as determined by ChIP-Seq, is plotted. Besides centromere (*cen*), telomere (*tel*), and mating type (*mat*) locus, H3K9me2 peaks distribute across the genome. (B) H3K9me2 distribution is shown at individual loci. Chromosome positions in (A) and (B) correspond to Sanger Center pombe database 2015 assembly. (C) H3K9me2 enrichment DNA reads in three biological wild type cells were plotted over sub-telomeric region of all three chromosomes. Chromosome coordinates are indicated above the peaks. Scale bars on the right denote H3K9me2 enrichment DNA reads numbers normalized per one million reads.
(TIF)

S7 Fig HU promotes spreading of heterochromatin across the boundaries of the silent domain at mating type locus. (A) Diagram illustrates the genetic construct at the mating type locus of strain PSG552, carrying *ade6*^+^ and *ura4*^+^ transgenes flanking the silent domain (green box). (B) HU induces transgenes silencing. Cells are pre-treated with 0mM or 2mM HU for 24 hours then spread on YE+4S+5-FOA or YE+4S+5-FOA+2mMHU, respectively, and incubated at 25^°^C for 7 days. The 5-FOA resistance colonies in white or red color are counted. (C) Stability of the silenced states of the transgenes. Cell suspension of five different 5-FOA-resistent (*IRR*::*ura4*^+^-silent) colonies are plated on the YE+4S and YE+4S+5-FOA plates, and incubated at 25^°^C for 7 days. The total viable colonies and 5-FOA-resistent colonies are counted, and the percentages of *IRR*::*ura4*^+^-silent progeny are calculated. The error bars are 1 SD of percentages for four independent plates.
(TIF)

S8 Fig Enhanced spreading of heterochromatin results from the increased Atf1-mediated recruitment of Swi6. (A) The mRNA expression level of Atf1 is increased in *cdc22-3* mutant cells. Atf1 mRNA is detected using RT-PCR. ß-actin mRNA is used as an internal standard. And the relative ration of Atf1 mRNA in wild type is normalized to 1. (B) The mRNA expression level of Atf1 is decreased in *clr1D* cells and is slightly increased in *clr1D* cells with HU treatment. (C) The diagram illustrates the genetic construct of the mating type locus. Green box indicates a heterochromatin region. (D-E) The bindings of Swi6 (D) and Epe1 (E) at mating type boundaries and sub-telomeric region are analyzed by ChIP as well as qPCR. Levels of Swi6 or Epe1 are assayed by ChIP as well as qPCR from *cdc22-3* and wild type strains. The PCR primer in sub-telomeric region is in the chromosome 1 left arm end. Primers of *tel-1* is in the chromosome 1 20000-25000bp, and *tel-2* is in the chromosome 1 15000-20000bp. The primers chosen here is according to the previous study [60]. Recovery ratios of immuno-precipitated DNA to total DNA at the indicated loci are normalized to the value at *actin* locus. The error bars are 1 SD of percentages for three replicates.
(TIF)

S1 Table List of yeast strains used in this study. (DOCX)
