# Supplementary figures and images for: Replication stress affects the fidelity of nucleosome-mediated epigenetic inheritance
Source: PLoS Genet. 2017 Jul 27;13(7):e1006900. doi: 10.1371/journal.pgen.1006900 (PMC5549764; doi:10.1371/journal.pgen.1006900)

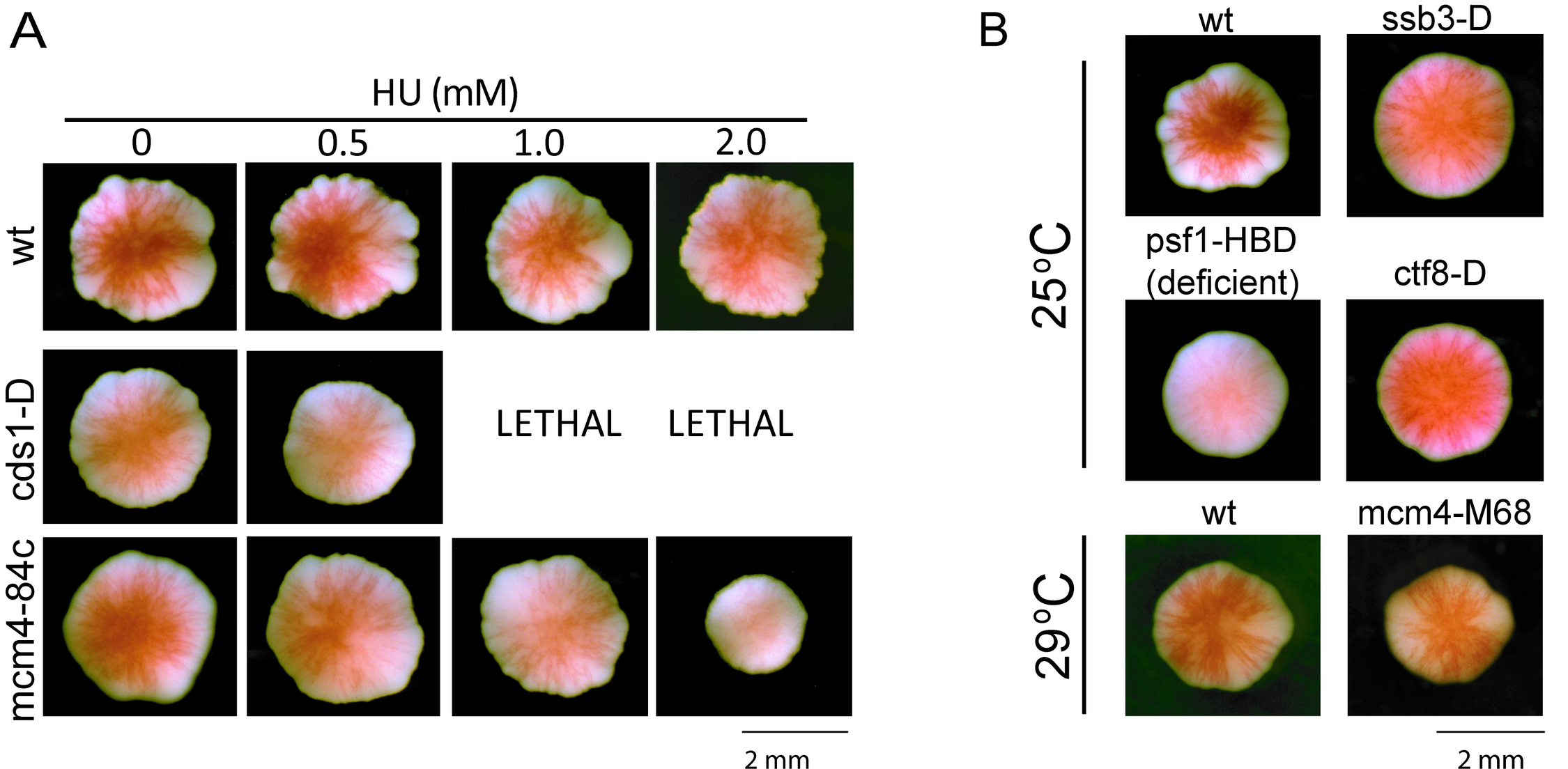

Supplement: S1 Fig — (A) cds1-D (LW21) and mcm4-84c (CY344) colonies exhibit enhanced sectoring at the presence of HU. Cells of the mutant strains and wild type (XL762) are planted at the density of one cell per cm2 on YE+4S plate containing HU as labeled, and incubated at 25°C for eight days. (B) High degrees of sectoring on the colonies of the replication mutants. Wild type, ctf8-D and ssb3-D cells are planted on YE+4S plates, and psf1-HBD cells on YE+4S+100nM β-estradiol and YE+4S+0.1nM β-estradiol, respectively, incubated at 25°C for eight days. Wild type and mcm4-M68 cells incubated at 29 oC for six days. One representative colony for each was shown. Scale bar is 2mm. (TIF) [file pgen.1006900.s001.tif]

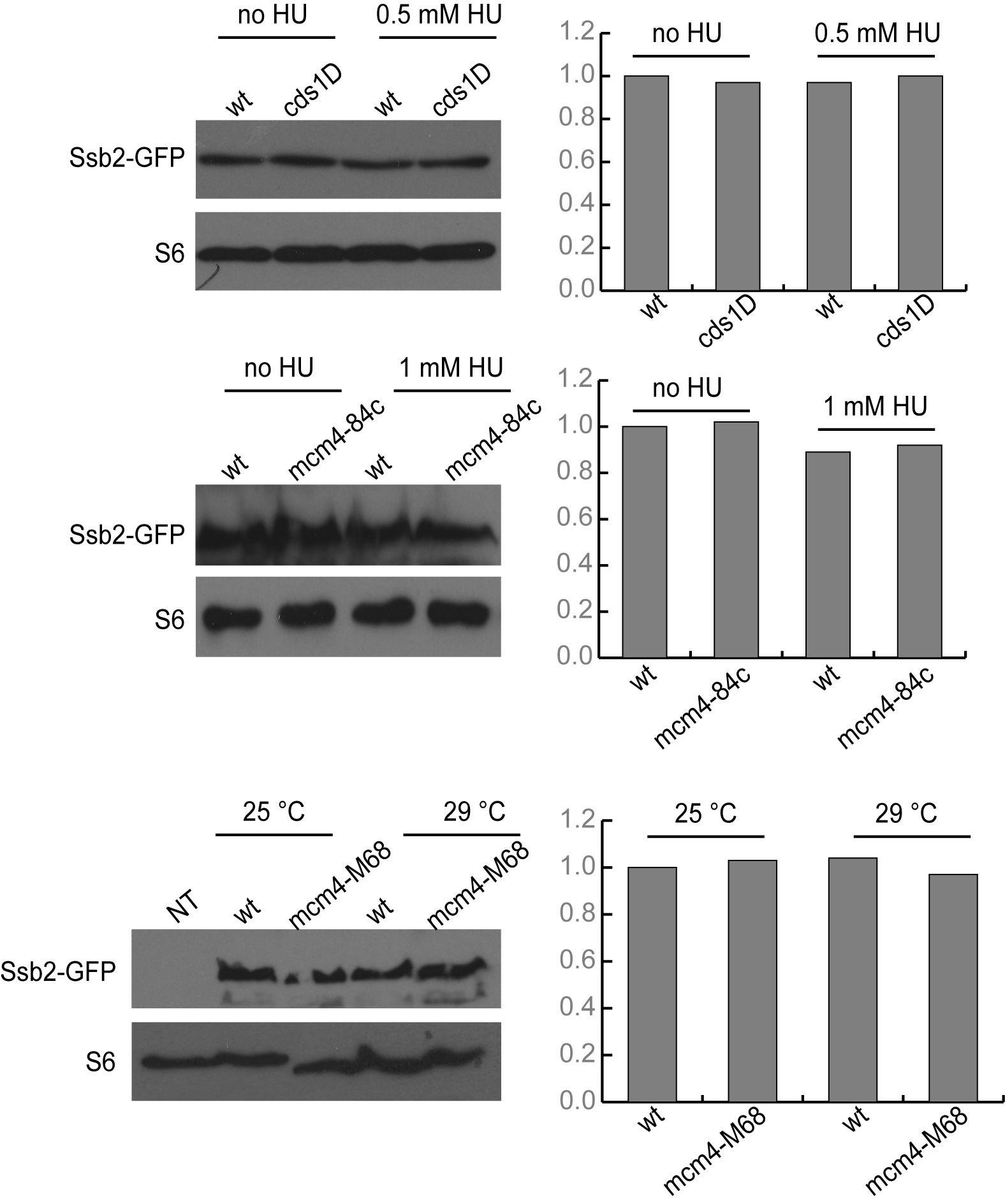

Supplement: S2 Fig — The protein levels of Ssb2-GFP are examined by western blot analysis, using ribosome protein S6 as a negative control. The gray value of each band is measured, and the ratios of GFP/S6 of the indicated strains were normalized to the value of GFP/S6 in wild type without HU treatment. (TIF) [file pgen.1006900.s002.tif]

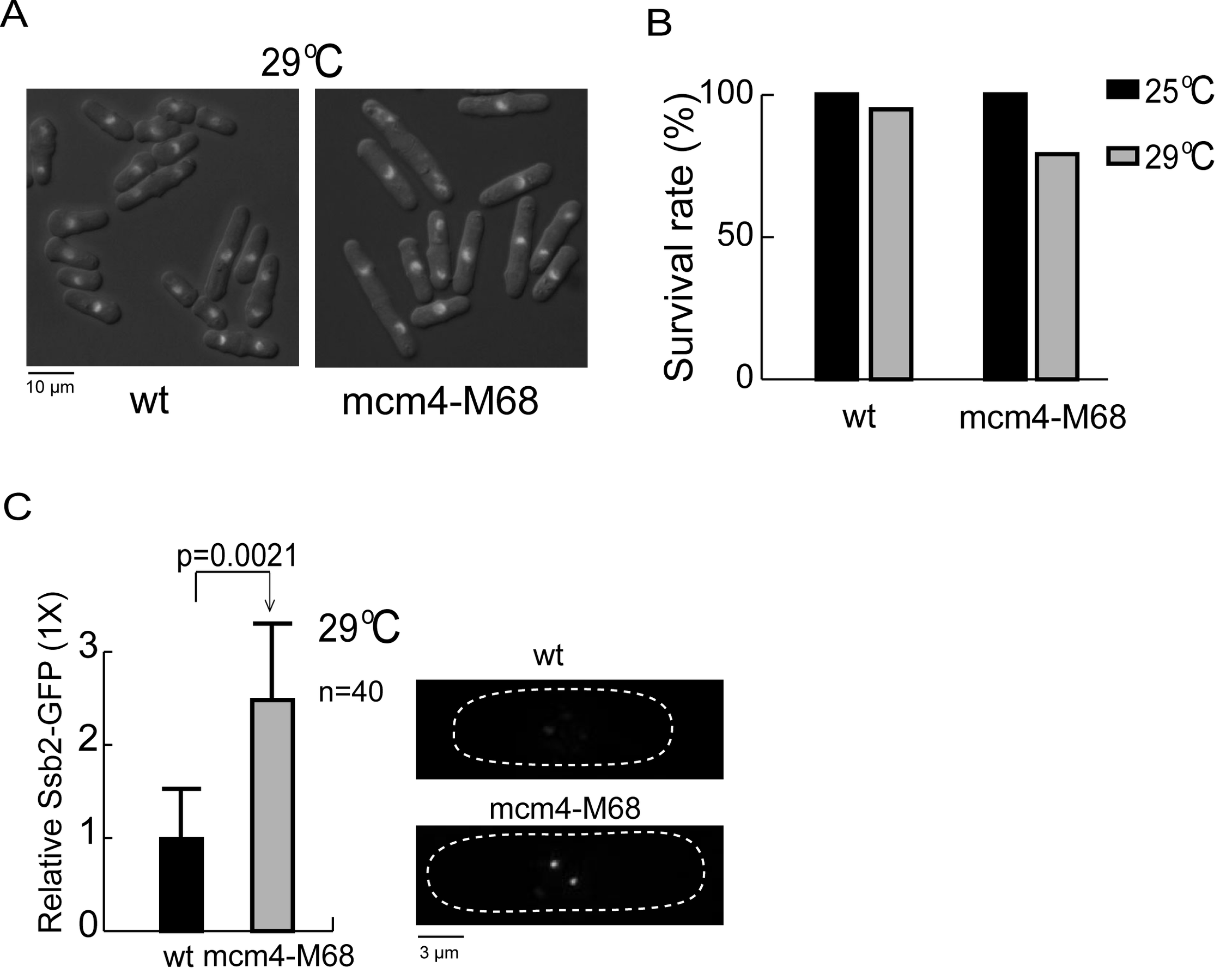

Supplement: S3 Fig — (A) mcm4-M68 (LW22) mutant cells show elongated cell shape compare with wild type cells at 29°C. Both mutant and wild type cells are grown at 25°C over night, then shift to 29°C for 6h. Cells are harvested and fixed with methanol. After washing with PBS, cells are stained with Hoechst dye for microscopic examining of the nuclear morphology. Scale bar is 10μm. (B) mcm4-M68 mutant cells exhibit mild reduced survival rate at 29°C. Cells are grown at 25°C over night. Cell suspensions of both strains are plated onto YE+5S solid media with 400 colonies per plate, and incubated at 25°C and 29°C separately. The number of survival colonies was counted. (C) Increased levels of Ssb2-GFP signal in the mutant G2 phase cells. Wild type, mcm4-M68 cells are grown in the liquid YE+5S media at 25°C over night, then shift to 29°C for 6h. Ssb2-GFP signal is measured as in Fig 2A. Representative G2 phase cell images are shown in the right panels. Scale bar is 3μm. (TIF) [file pgen.1006900.s003.tif]

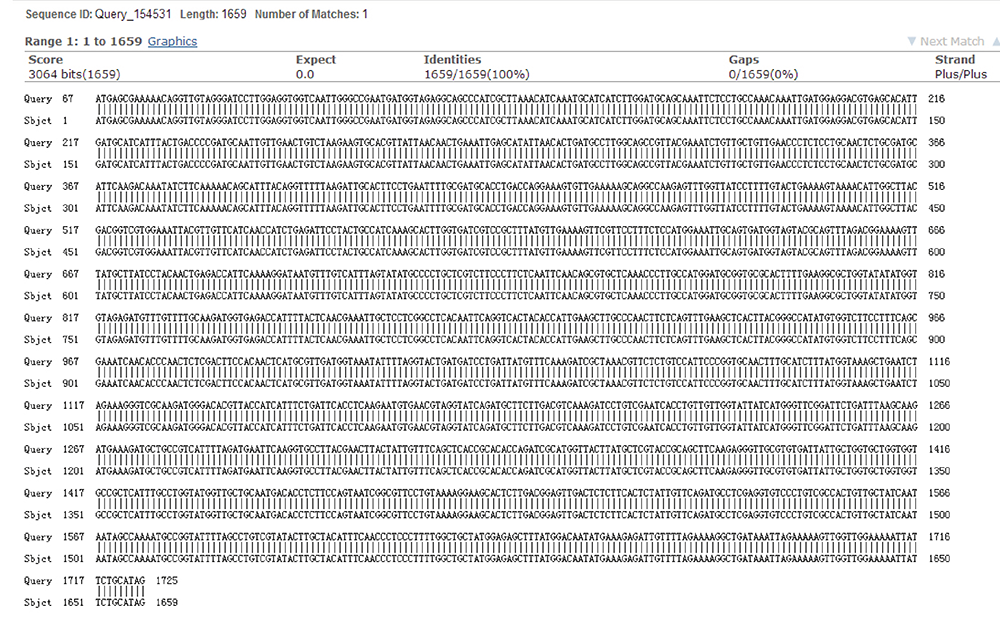

Supplement: S4 Fig — Four red colonies from L(BglII)::ade6+and ura4::cenH-ade6+ strains were picked and the ade6+ reporter was PCR amplified and subjected to sequencing. No mutation was found in ade6+ in these colonies. DNA sequence alignment for one of the four sequencing results is shown. Query: DNA sequence of ade6+ PCR product from red colonies. Subject: DNA sequence of ade6+ gene downloaded from website (http://www.pombase.org). (TIF) [file pgen.1006900.s004.tif]

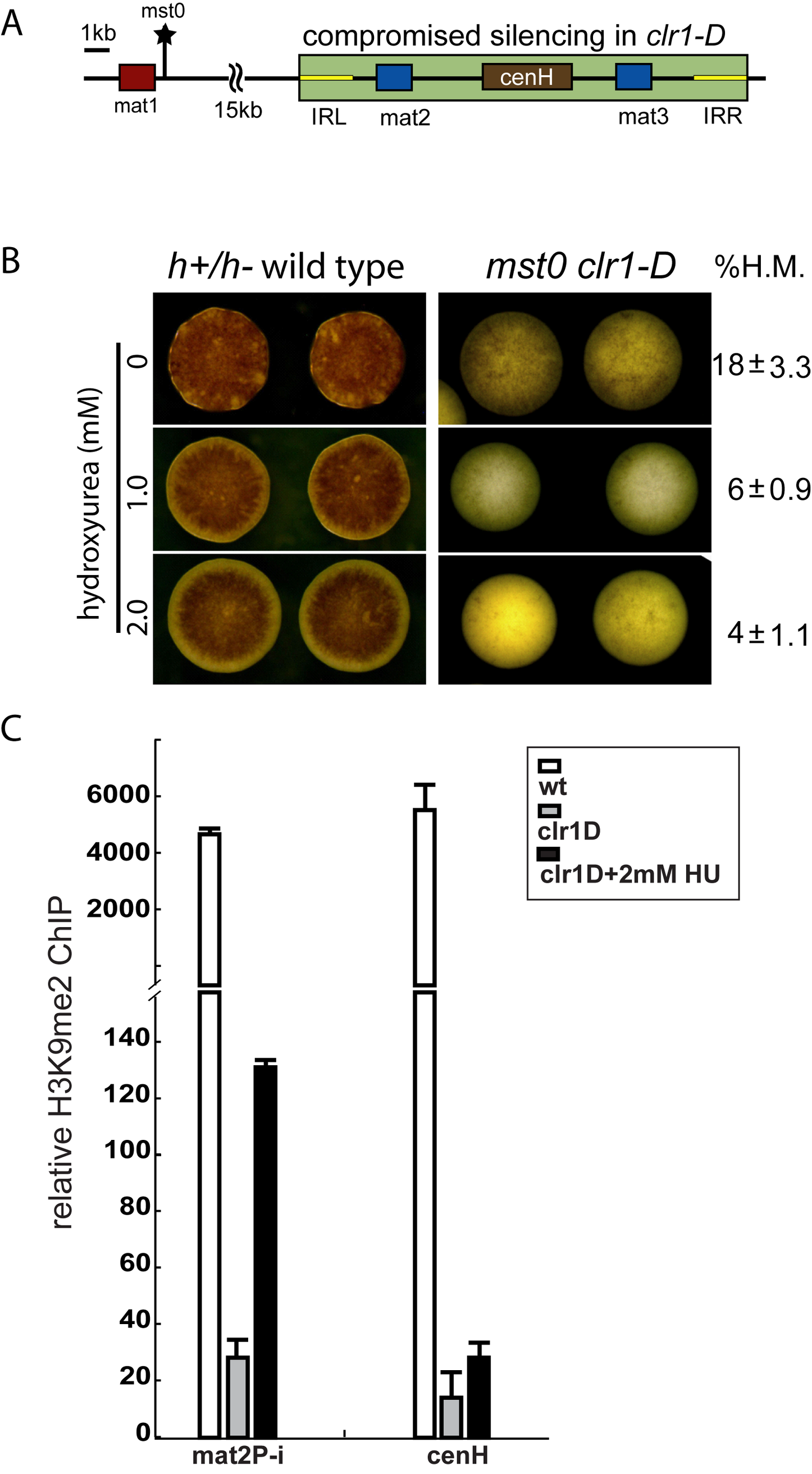

Supplement: S5 Fig — (A) The diagram illustrates the genetic construct of the mating type locus. Light green box indicates a compromised silencing domain. (B) HU treatment restores the silencing of mat2-P at the mating type locus in clr1-D strain. mat1-msmt0 clr1-D haploid cells (LW63) and h-/h+ wild type diploid cells (LW52) are plated on sporulation medium (EMM-N) containing HU as labeled, incubated at 30°C for five days, stained with iodine vapor. Two colonies each are shown. Cell suspensions of the colonies are then stained with Hoechst dye for microscopic examining of the nuclear morphology. The percentage of clr1-D cells showing a haploid meiosis phenotype (H.M.) is quantified in five independent colonies with >100 cells scored in each. (C) HU treatment promotes heterochromatin in clr1-D strain. mat1-msmt0 clr1-D haploid cells (LW63) were cultured in EMM5S liquid media until OD600 reached 0.1, then shifted to EMM-N liquid media with or without 2mM HU for 17 hours. Cells were harvested and immuno-precipitated with anti-H3K9me2 antibody. Heterochromatin enrichment was examined by ChIP using quantitative real-time PCR. Recovery ratios of immuno-precipitated DNA to total DNA at the indicated loci were normalized to the value of tubulin gene. Data are mean ±s.d. (n = 3). (TIF) [file pgen.1006900.s005.tif]

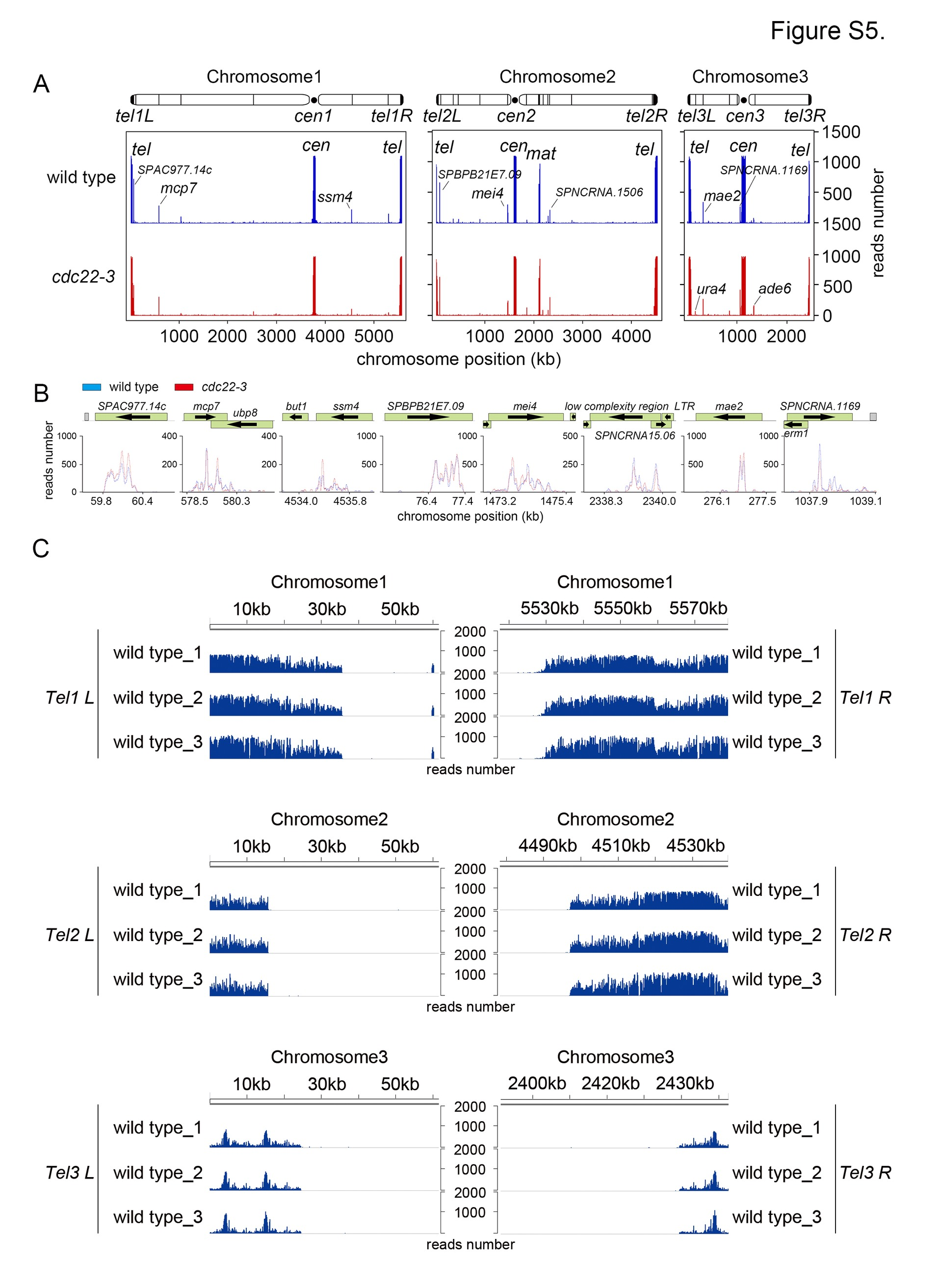

Supplement: S6 Fig — (A) Relative fold enrichment of dimethylated H3K9 (H3K9me2), as determined by ChIP-Seq, is plotted. Besides centromere (cen), telomere (tel), and mating type (mat) locus, H3K9me2 peaks distribute across the genome. (B) H3K9me2 distribution is shown at individual loci. Chromosome positions in (A) and (B) correspond to Sanger Center pombe database 2015 assembly. (C) H3K9me2 enrichment DNA reads in three biological wild type cells were plotted over sub-telomeric region of all three chromosomes. Chromosome coordinates are indicated above the peaks. Scale bars on the right denote H3K9me2 enrichment DNA reads numbers normalized per one million reads. (TIF) [file pgen.1006900.s006.tif]

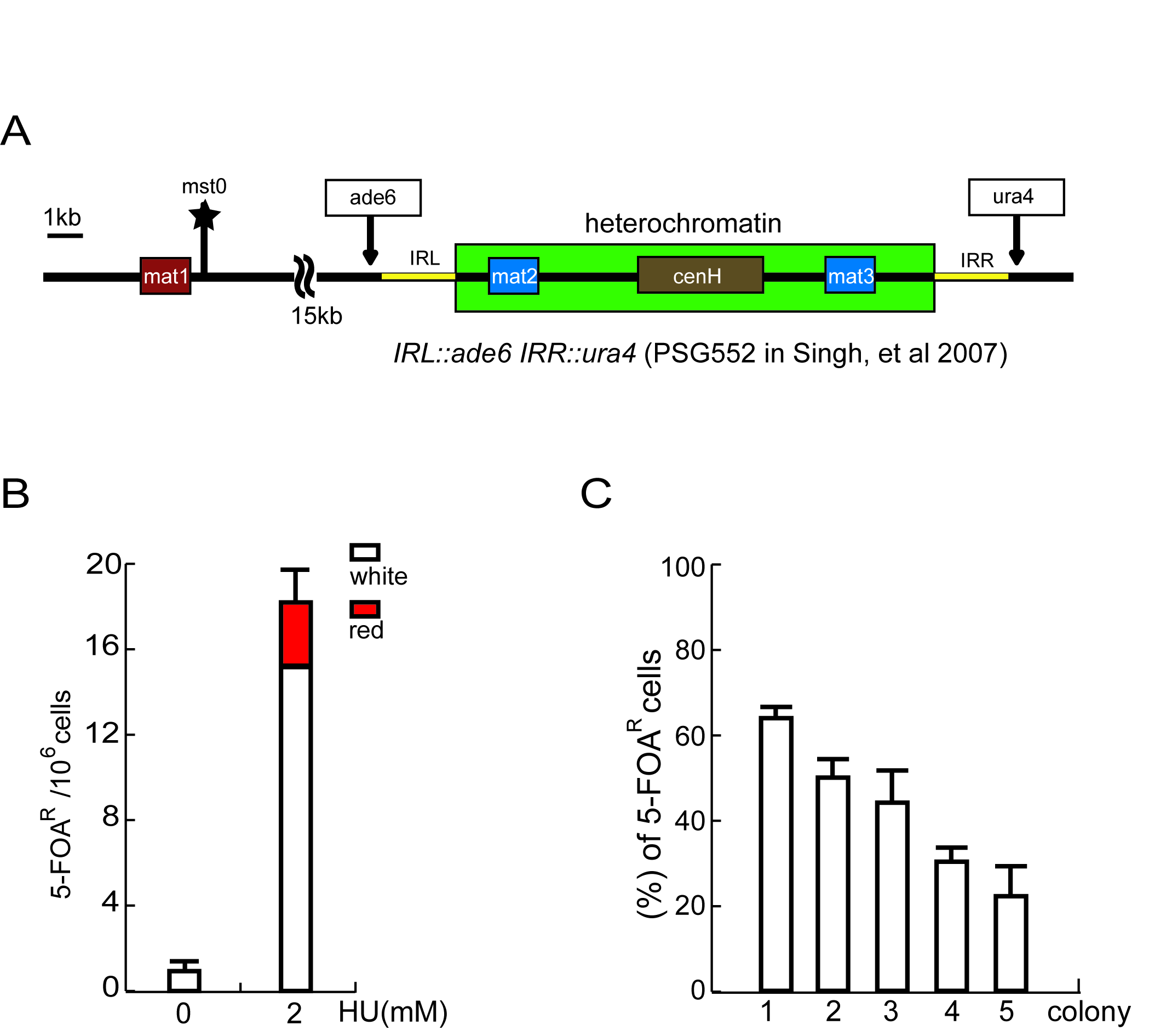

Supplement: S7 Fig — (A) Diagram illustrates the genetic construct at the mating type locus of strain PSG552, carrying ade6+ and ura4+ transgenes flanking the silent domain (green box). (B) HU induces transgenes silencing. Cells are pre-treated with 0mM or 2mM HU for 24 hours then spread on YE+4S+5-FOA or YE+4S+5-FOA+2mMHU, respectively, and incubated at 25°C for 7 days. The 5-FOA resistance colonies in white or red color are counted. (C) Stability of the silenced states of the transgenes. Cell suspension of five different 5-FOA-resistent (IRR::ura4+-silent) colonies are plated on the YE+4S and YE+4S+5-FOA plates, and incubated at 25°C for 7 days. The total viable colonies and 5-FOA-resistent colonies are counted, and the percentages of IRR::ura4+-silent progeny are calculated. The error bars are 1 SD of percentages for four independent plates. (TIF) [file pgen.1006900.s007.tif]

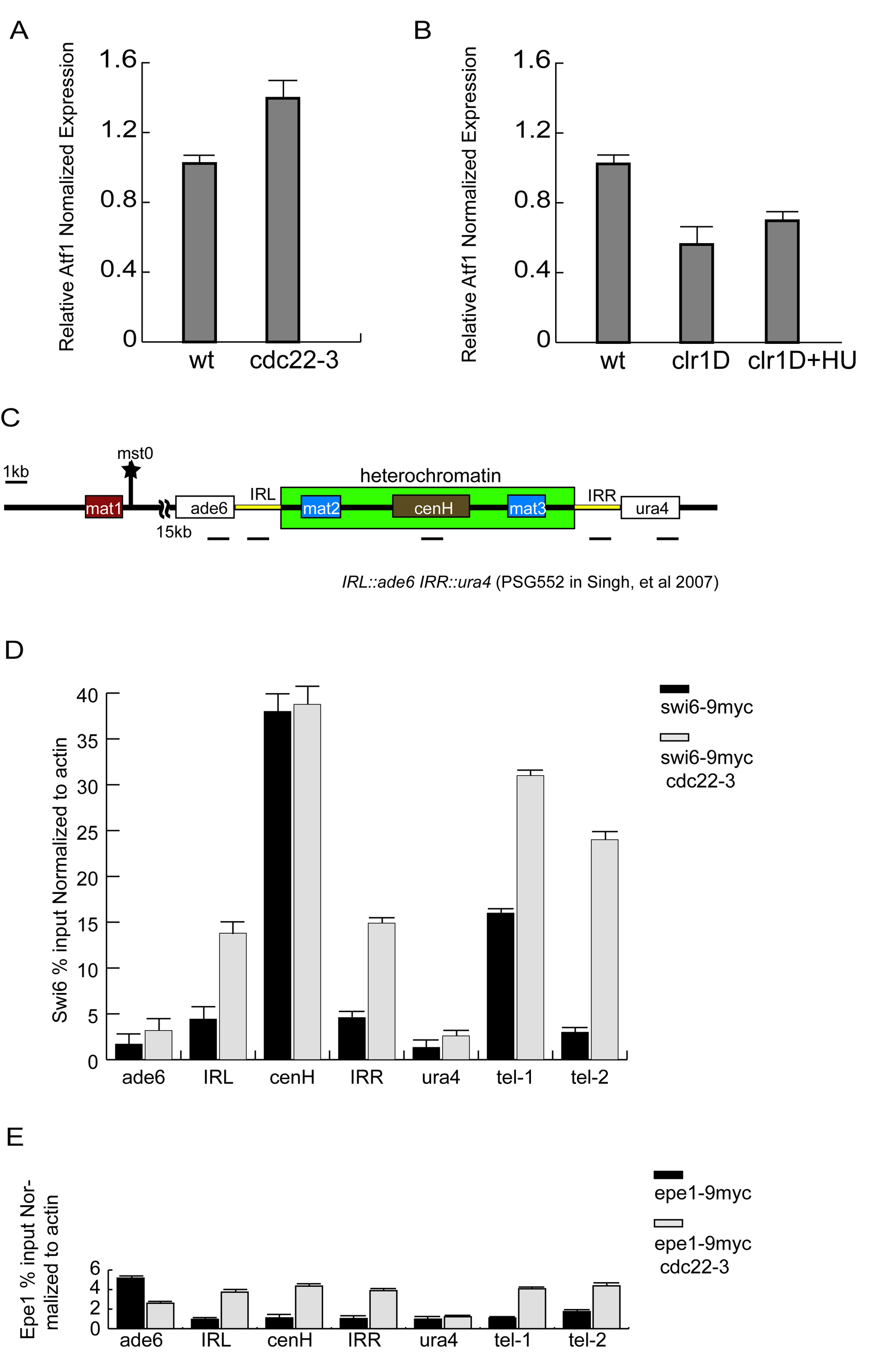

Supplement: S8 Fig — (A) The mRNA expression level of Atf1 is increased in cdc22-3 mutant cells. Atf1 mRNA is detected using RT-PCR. ß-actin mRNA is used as an internal standard. And the relative ration of Atf1 mRNA in wild type is normalized to 1. (B) The mRNA expression level of Atf1 is decreased in clr1D cells and is slightly increased in clr1D cells with HU treatment. (C) The diagram illustrates the genetic construct of the mating type locus. Green box indicates a heterochromatin region. (D-E) The bindings of Swi6 (D) and Epe1 (E) at mating type boundaries and sub-telomeric region are analyzed by ChIP as well as qPCR. Levels of Swi6 or Epe1 are assayed by ChIP as well as qPCR from cdc22-3 and wild type strains. The PCR primer in sub-telomeric region is in the chromosome 1 left arm end. Primers of tel-1 is in the chromosome 1 20000-25000bp, and tel-2 is in the chromosome 1 15000-20000bp. The primers chosen here is according to the previous study [60]. Recovery ratios of immuno-precipitated DNA to total DNA at the indicated loci are normalized to the value at actin locus. The error bars are 1 SD of percentages for three replicates. (TIF) [file pgen.1006900.s008.tif]
