# Supplementary material for: Replication stress affects the fidelity of nucleosome-mediated epigenetic inheritance
Source: PLoS Genet. 2017 Jul 27;13(7):e1006900. doi: 10.1371/journal.pgen.1006900 (PMC5549764; doi:10.1371/journal.pgen.1006900)
Supplement: S1 Table — (DOCX) [file pgen.1006900.s009.docx]

Table S1. Yeast strains used in this study.

| Name | Genotype | Source |
| --- | --- | --- |
| LW5 | *h^90^ ssb2-GFP-HA::kanR ade6-210 leu1-32 ura4-D18 ade6-216 lys1-131* | YGRC |
| LW17  LW18 | *ssb2-GFP-HA::kanR mcm4-84c ::kanR leu1-32 ura4-D18 ade6-216 lys1-131*  *h^-^ mcm4-M68 (cdc21-M68) ts imrL::ade6^+^ ade6::kanR* | This study  This study |
| LW21  LW22 | *h^+^ mcm4-84c::kanR imrL ::ade6^+^ ade6::kanR*  *ssb2::ssb2-GFP-HA::kanR mcm4-M68 (cdc21-M68) ts leu1-32 ura4-D18 ade6-216 lys1-131* | This study  This study |
| LW37 | *psf1-HBD-kanMX ade6::kanR imrL::ade6^+^* | This study |
| LW45 | *mat1-msmt0 leu1-32 ade6DN/N* | This study |
| LW49 | *psf1-HBD-kanMX ssb2-GFP-HA::kanR ade6-210* | This study |
| LW52 | *h^+^/h^-^ diploid* | Lab stock |
| LW53 | *cds1*∆*::ura4^+^ ssb2-GFP-HA ::kanR leu1-32 ura4-D18 ade6 ? lys1 ?* | This study |
| LW54 | *ssb3*∆*::kanR ssb2-GFP-HA ::kanR leu1-32 ura4-D18 ade6 ? lys1 ?* | This study |
| LW56 | *ctf8*∆*::kanR ssb2-GFP-HA ::kanR leu1-32 ura4-D18 ade6 ? lys1 ?* | This study |
| LW63  LW75  LW339  LW341  LW346  LW348 | *mat-msmt0 clr1*∆*::kanR ade6::natR*  *mat1-msmt0 ura4::CenH-ade6^+^ leu1-32 ade6DN/N*  *mat-msmt0 epe1-9myc ::hph*  *mat-msmt0 epe1-9myc ::hph cdc22-3*  *mat-msmt0 swi6-9myc ::hph*  *mat-msmt0 swi6-9myc ::hph cdc22-3* | This study  This study  This study  This study  This study  This study |
| AP144 | *mat1-msmt0 L (Bgl* I I*)::ade6^+^ leu1-32 ura4-D18 ade6-210 his2-* | Cohen’s Lab |
| CY9 | *ctf8*∆*::kanR imrL ::ade6^+^ ade6::natR* | Lab stock |
| CY228 | *ssb3*∆*::kanR imrL ::ade6^+^ ade6::natR* | Lab stock |
| CY344  PSG552 | *cds1*∆*::kanR imrL ::ade6^+^ ade6::natR*  *mat1-msmt0 IRL::ade6 IRR::ura4 leu1-32 ura4-D18 ade6Δ-1* | Lab stock  Klar’s Lab |
| XL762 | *h^+^ imrL ::ade6^+^ ade6::kanR* | Lab stock |
